# Vegan Diet Is Associated With Favorable Effects on the Metabolic Performance of Intestinal Microbiota: A Cross-Sectional Multi-Omics Study

**DOI:** 10.3389/fnut.2021.783302

**Published:** 2022-01-07

**Authors:** Magdalena Prochazkova, Eva Budinska, Marek Kuzma, Helena Pelantova, Jaromir Hradecky, Marie Heczkova, Nikola Daskova, Miriam Bratova, Istvan Modos, Petra Videnska, Petra Splichalova, Solomon A. Sowah, Maria Kralova, Marina Henikova, Eliska Selinger, Krystof Klima, Karel Chalupsky, Radislav Sedlacek, Rikard Landberg, Tilman Kühn, Jan Gojda, Monika Cahova

**Affiliations:** ^1^Department of Internal Medicine, Kralovske Vinohrady University Hospital and Third Faculty of Medicine, Charles University, Prague, Czechia; ^2^Research Centre for Toxic Compounds in the Environment, Faculty of Science, Masaryk University, Brno, Czechia; ^3^Laboratory of Molecular Structure Characterization, Institute of Microbiology, Czech Academy of Sciences, Prague, Czechia; ^4^Faculty of Forestry and Wood Sciences, Czech University of Life Sciences, Prague, Czechia; ^5^Center of Experimental Medicine, Institute for Clinical and Experimental Medicine, Prague, Czechia; ^6^First Faculty of Medicine, Charles University, Prague, Czechia; ^7^Division of Cancer Epidemiology, German Cancer Research Center (DKFZ), Heidelberg, Germany; ^8^Medical Faculty and University Hospital, Heidelberg University, Heidelberg, Germany; ^9^Department of Applied Mathematics and Computer Science, Masaryk University, Brno, Czechia; ^10^Czech Centre for Phenogenomics, Institute of Molecular Genetics, Czech Academy of Sciences, Prague, Czechia; ^11^Division of Food and Nutrition Science, Department of Biology and Biological Engineering, Chalmers University of Technology, Goteborg, Sweden; ^12^Institute of Global Food Security, Queen's University Belfast, Belfast, United Kingdom; ^13^Heidelberg Institute of Global Health (HIGH), Medical Faculty and University Hospital, Heidelberg University, Heidelberg, Germany

**Keywords:** vegan diet, omics signature, protein fermentation, short-chain fatty acids (SCFAs), metabolic health

## Abstract

**Background and Aim:** Plant-based diets are associated with potential health benefits, but the contribution of gut microbiota remains to be clarified. We aimed to identify differences in key features of microbiome composition and function with relevance to metabolic health in individuals adhering to a vegan vs. omnivore diet.

**Methods:** This cross-sectional study involved lean, healthy vegans (*n* = 62) and omnivore (*n* = 33) subjects. We assessed their glucose and lipid metabolism and employed an integrated multi-omics approach (16S rRNA sequencing, metabolomics profiling) to compare dietary intake, metabolic health, gut microbiome, and fecal, serum, and urine metabolomes.

**Results:** The vegans had more favorable glucose and lipid homeostasis profiles than the omnivores. Long-term reported adherence to a vegan diet affected only 14.8% of all detected bacterial genera in fecal microbiome. However, significant differences in vegan and omnivore metabolomes were observed. In feces, 43.3% of all identified metabolites were significantly different between the vegans and omnivores, such as amino acid fermentation products p-cresol, scatole, indole, methional (lower in the vegans), and polysaccharide fermentation product short- and medium-chain fatty acids (SCFAs, MCFAs), and their derivatives (higher in the vegans). Vegan serum metabolome differed markedly from the omnivores (55.8% of all metabolites), especially in amino acid composition, such as low BCAAs, high SCFAs (formic-, acetic-, propionic-, butyric acids), and dimethylsulfone, the latter two being potential host microbiome co-metabolites. Using a machine-learning approach, we tested the discriminative power of each dataset. Best results were obtained for serum metabolome (accuracy rate 91.6%).

**Conclusion:** While only small differences in the gut microbiota were found between the groups, their metabolic activity differed substantially. In particular, we observed a significantly different abundance of fermentation products associated with protein and carbohydrate intakes in the vegans. Vegans had significantly lower abundances of potentially harmful (such as p-cresol, lithocholic acid, BCAAs, aromatic compounds, etc.) and higher occurrence of potentially beneficial metabolites (SCFAs and their derivatives).

## Introduction

Recent studies suggest that the composition and function of the gut microbiome play a fundamental role in the development of non-communicable diseases (1). Diet is a key determinant of the relationship between humans and their microbial residents, as it affects the composition of gut microbial ecosystem, which, in turn, impacts on human physiology *via* direct interaction with the immune system and metabolic outputs (2). Adherence to plant-based diets (vegetarian or vegan) was shown to be associated with potential health benefits (3). Epidemiological studies show a lower incidence of several chronic diseases, such as type 2 diabetes (T2D), cardiovascular diseases, and cancer (4–7). When compared to lacto-ovo-vegetarian diets, vegan diets may lower the risk of obesity, hypertension, T2D, and cardiovascular mortality. Moreover, with respect to certain cancers, a strict vegan diet may be more beneficial than a lacto-ovo-vegetarian one, although further studies are needed (8, 9). Intervention studies comparing vegan or vegetarian diets vs. omnivorous diets have shown beneficial effects of these diets on cardiometabolic risk factors (10), T2D (11), and obesity (12).

Whether the beneficial effects of plant-based diets can be attributed to their nutritional composition alone or whether they are mediated, at least partly, by different microbiota and their metabolites remains to be clarified. There is no clear consensus concerning the effect of a profound dietary switch to a strict plant-based diet on gut microbiota, microbial fermentation products, and their impact on host metabolism. This issue gains in importance with the increasing interest in microbiota manipulation in the therapy of noncommunicable diseases, and with the simultaneous trend for plant-based diets (13).

Given the association among diet, gut microbiome/metabolome (MIME), and metabolic health, we hypothesized that there are specific compositional and functional characteristics of MIME that link different eating habits to metabolic phenotype. To this end, we explored two metabolically healthy groups defined by distinct dietary habits, i.e., lean healthy long-term vegans (i.e., those who have been adhering to a vegan diet for at least 3 years) and lean healthy omnivores. Our aim was to analyze the fecal microbiome as well as serum, urine, and fecal metabolomes of these groups in order to identify key features associated with the different diets and provide potential functional links among them.

## Materials and Methods

### Study Population

Sixty-two self-reported vegans (VGs) and 33 omnivores (Os) were screened and enrolled between October 2018 and October 2019 for cross-sectional comparison. The VGs strictly avoided all animal products for at least 3 years, and the omnivore group comprised subjects without any dietary restrictions who consumed meat and other animal products on a daily basis. In both groups, the exclusion criteria were age under 18 years, obesity defined as BMI > 30, chronic diseases related to metabolism, diseases of the digestive tract, antibiotic therapy in the past 3 months, pregnancy, any chronic medication (excluding hormonal contraception), and regular alcohol consumption defined as any alcoholic drink on a daily basis. A clinical visit was scheduled after enrollment. After 12-h overnight fast, blood and urine were sampled, and clinical examination was performed. Afterward, oral glucose tolerance test (OGTT, 75g glucose) was performed with blood sampling at 0, 30, 60, 90, and 120 min. All blood samples were immediately centrifuged and snap frozen at −80°C before analyses. Glucose homeostasis indices were derived from serum glucose and insulin changes in OGTT: AUCs for glucose and insulin using trapezoid rule, Matsuda index of insulin sensitivity as described elsewhere (14).

### Study Approval

All the participants signed informed consent prior to enrollment. The research protocol was approved by the Ethics Committee of the Third Faculty of Medicine of the Charles University and the Ethics Committee of University Hospital Kralovske Vinohrady (EK-VP/26/0/2017) in accordance with the Declaration of Helsinki.

### Dietary Intake Assessment

Dietary records and stool samples were obtained no longer than a week after the clinical visit. A 3-day prospective record supervised by a trained dietitian was used to assess the macronutrient composition and fiber content of the diet. Each participant filled in a prospective record, where dietary data from 3 typical days were collected (2 working days, 1 weekend day). The volunteers were educated. Instructions were given for portion size estimation and recording of foods in sufficient detail to obtain an accurate estimate of consumed portions, and a portion estimation guide was given as a reference. Moreover, examples of complete and incomplete diaries were explained to show how to appropriately record the intake. After collection, the records were retrospectively checked by an independent researcher. The USDA database was used for assessment of food composition, NutriServis PROFI, and CR, and a program was used for dietary intake calculations. Daily intake of carbohydrates, lipids, proteins, and dietary fiber was calculated separately.

### Fecal Sample Collection, Storage, and Processing

Fecal samples collected at home had been immediately stored at −20°C until transported in the frozen state to the laboratory within 7 days of collection. Once thawed on ice, the samples were homogenized using stomacher (BioPro, Czechia); one aliquot was used for DNA extraction, one aliquot was used for dry mass estimation, and the rest was aliquoted and stored at −50°C. For metabolome analyses, the aliquots were thawed and diluted with sterile water to 1% dry mass equivalent. For bile acid composition analyses, the samples were lyophilized.

### Gut Microbiome Analysis

DNA from the fecal samples was isolated with QIAmp PowerFecal DNA Kit (Qiagen, Germany), and the V4 region of the bacterial 16S rRNA gene was amplified by PCR. A library was prepared according to the Illumina 16S Metagenomic sequencing Library Preparation protocol with some deviations described below. Each PCR was performed with an EMP primer pair consisting of Illumina overhang nucleotide sequences, an inner tag, and gene-specific sequences. The sequences of EMP primers, overhang, and tag sequences are shown in Supplementary Table 1. The Illumina overhang served to ligate the Illumina index and adapter. Each inner tag, i.e., a unique sequence of 7–9 bp, was designed to differentiate the samples into groups. The total reaction volume of PCR was 30 μl, and cycling parameters included initial denaturation at 98°C for 30 s, followed by 30 cycles of 10 s denaturation at 98°C, 15 s annealing at 55°C and 30-s extension at 72°C, followed by final extension at 72°C for 2 min. Samples with different inner tags were equimolarly pooled, and pools were used as a template for second PCR with Nextera XT (Illumina, United States) indexes. Differently indexed samples were equimolarly pooled. The final library was diluted to a concentration of 8 pM, and 20% of PhiX DNA (Illumina, United States) was added. Sequencing was performed with the Miseq reagent kit V2 using a MiSeq instrument according to the manufacturer's instructions (Illumina, Hayward, CA, United States). Raw sequences were processed using an in-house pipeline based on a DADA2 amplicon denoiser (15), and a standard bioinformatic procedures within the QIIME 1.9.1 package (16).

### Availability of Materials

Sequencing data are available in the European Nucleotide Archive database under the accession number PRJEB43938. Publication of our dietary data, as well metabolomics data, was not possible, as it was not covered by the participants' informed consent used for the study. However, pseudonomized data will be made available by the corresponding authors upon reasonable request.

### Determination of *But* Gene Expression

The abundance of butyryl-CoA:acetate CoA-transferase (*but*) gene in the DNA isolated from stool samples was determined, as described in Daskova et al. (17) Briefly, bacteria containing the *but* gene in their genome were identified using FunGene Database. The selection was narrowed only to bacteria already found in human gut microbiota. The sequence of *but* gene coding for butyryl-CoA:acetate CoA-transferase is highly variable among gut butyrate producers;, therefore, degenerate primers targeting different variants of the *but* gene were designed. Even when degenerate primers were used, six different primer pairs had to be designed in order to cover all *but* gene sequences (17) (Supplementary Table 2). The copy number of *but* gene in the DNA isolated from the stool samples was determined by quantitative PCR (qPCR) and normalized to spike DNA (*C. elegans* UNC-6 gene; forward primer GAAGAGCAAGATCAGTGTTC, reverse primer CTTGCAAATGACACCTTG).

### Short-Chain Fatty Acid in Plasma

Short-chained fatty acids (SCFAs) were analyzed in the plasma by LC-MS according to a method described before (18). Standards for SCFAs used were: formic acid (C1) (Scharlau, Spain), acetic acid (C2) (Honeywell, United States), propionic acid (C3) (Alfa Aesar, United States), butyric acid (C4) (Sigma Aldrich, United States), isobutyric acid (C4) (Alfa Aesar, United States), succinic acid (C4) (Acros, United States), isovaleric acid (C5) (Sigma Aldrich, United States), valeric acid (C5) (Alfa Aesar, United States), and caproic acid (C6) (Sigma Aldrich, United States). Analytical reagent-grade 3-nitrophenylhydrazine (3NPH)-HCl (97%), 2-nitrophenylhydrazine N-(3-dimethylaminopropyl)-N0-ethylcarbodiimide (EDC) HCl, quinic acid, HPLC-grade pyridine, and Lichrosol reagent-grade MeOH and water were obtained from Sigma–Aldrich. Acetonitrile Optima LCMS Grade was obtained from Thermo Fisher Scientific (United States). ^13^C6-3NPH-HCl was custom synthesized to us by IsoSciences Inc. (King of Prussia, PA, United States) (catalouge 13309). This custom-synthesized compound was structurally confirmed by 1H NMR spectroscopy and by MS/MS on a triple-quadruple mass spectrometer.

### Volatile Compound (VOC) Analysis on Feces

Volatile fingerprinting of the fecal samples was performed using an Agilent 7890B (Agilent Technologies, United States) gas chromatograph coupled to a Pegasus 4D (LECO, United States) time of flight mass spectrometer. Volatiles were collected using a solid-phase microextraction (SPME) fiber with divinylbenzene/carboxen/polydimethylsiloxane coating from Supelco (United States). Data acquisition and initial data processing were carried out using instrumental SW ChtomaTOF by LECO (United States).

### NMR Analyses

Analyses were performed on fecal extracts prepared from homogenized stool aliquot corresponding to 1% of dry mass. All the samples were measured on a 600 MHz Bruker Avance III (Bruker BioSpin, Rheinstetten, Germany) spectrometer equipped with a 5-mm TCI cryogenic probe head. 1D-NOESY, CPMG, and *J*-resolved experiments were performed using standard manufacturers' software Topspin 3.5. Concentrations of individual metabolites, identified by comparison of proton and carbon chemical shift with HMDB database, were expressed as PQN-normalized intensities of corresponding signals in 1D-NOESY (urine), CPMG (serum extracts), and 1D projections of *J*-resolved (fecal extracts) spectra. The list of quantified metabolites in the urine, serum, and fecal extracts with corresponding ^1^H and ^13^C chemical shifts is given in Supplementary Table 3. The representative ^1^H NMR spectra are shown in Supplementary Figures 1–3.

### Bile Acid Analysis on Feces

Methanol extract was prepared from lyophilized fecal homogenate (1 ml of 1% homogenate). Liquid chromatography separation of the extracts was performed using 1290 Infinity LC (Agilent Technologies, United States) followed by mass spectrometry using 6550 iFunnel LCQ- TOF-MS (Agilent Technologies, United States) equipped with a Dual AJS ESI probe in negative-ion mode. System control and data acquisition were performed with Agilent MassHunter Quadrupole Time of Flight Acquisition Software (B.06) with Qualitative Analysis (B.07 SP2) Software.

### Statistics

Statistical analyses were performed in R software packages and in-house scripts (19). For individual tasks, the following R packages were used: composition (clr transformation), zCompositions (zero multiplicative replacement) vegan (PERMANOVA), ropls (PLS-DA metrics), mixOmics (VIP identification, 2D score plot PLS-DA), effsize (Cliffs delta), and caret (machine learning library). Clinical characteristics of the observational sample were compared by standard tests. The microbiome and VOCs data were treated as compositional (proportions of total read count in each sample, non-rarefied or proportion of total area under curve), and prior to all the statistical analyses were transformed by centered log-ratio (clr) transformation, and zero values were handled using count using R package zCompositions. According to their abundance and prevalence, the bacteria were classified as “core microbial taxa” when they fulfilled the following conditions, i.e., abundance > 0.1% and prevalence > 75% at least in one experimental group. Other microbial taxa were classified as rare. NMR data were normalized by probabilistic quotient normalization (PQN). All the data were scaled (z-score) before applying PERMANOVA, PCA, PLS-DA, or random forest method. The genera were filtered by minimal prevalence, i.e., present at least in three samples per cohort, with minimum of nine reads each. Principal component analysis (PCA) was performed to investigate possible sample clustering in each dataset. For each data type, multivariable statistics (PERMANOVA) were applied to test the differences between the groups; for gut microbiome, PERMANOVA was performed on each of the five taxonomy levels (phylum, class, order, family, and genus) separately. Univariable statistical analyses were performed by Mann-Whitney-Wilcoxon test. The results were adjusted for multiple-hypothesis testing by Benjamini-Hochberg procedure with a cut-off level of false discovery rate equal to 0.1. A multivariable statistic evaluation was performed by partial least square discriminant analysis (PLS-DA). We analyzed the discriminating power of each omics dataset using machine learning; specifically, we used a random forest method. The validity of a model was verified by permutation test with 300 repetitions. Correlation networks based on Spearman's correlation coefficient were used to assess the correlation between the studied variables.

All methods are described in detail in Supplemental Experimental Procedures.

## Results

### Subject Characteristics

Clinical characteristics of the study participants are given in Table 1. Dietary consumption quantified using 3-day prospective dietary records showed no significant difference in total energy intake between the groups. In the omnivores, higher daily intake of protein and lipids was recorded, while the vegans' diet consisted of more carbohydrates and dietary fiber. We observed more favorable indices of glucose homeostasis, i.e., lower concentration of glycated hemoglobin and lower secretion of insulin during OGTT in the vegans. Regarding lipid metabolism, the vegans had lower plasma levels of total as well as LDL- and HDL-cholesterol. Serum CRP, which serves as an inflammatory marker, was significantly lower in the vegans, albeit in both groups it remained within physiological range. Fecal pH was significantly lower in the vegans, whose stool samples contained more water.

**Table 1:** Group characteristics for vegans and omnivores.

	**Omnivore**	**Vegan**	***p*-value**
**General characteristics**			
Sex [F/M]	17/16	25/37	
Weight [kg]	73.0 (24.4)	67.9 (16.6)	n.s.
Age [years]	31.3 (11.2)	30.9 (10.5)	n.s.
BMI [kg/m2]	22.8 (4.4)	21.6 (3.6)	n.s.
WHR	0.8 (0.1)	0.8 (0.1)	n.s.
**Body composition**			
Fat [kg]	13.9 (5.8)	11.6 (9.3)	n.s.
FFM [kg]	54.2 (23.4)	57.1 (19.3)	n.s.
TBW [kg]	39.7 (17.1)	41.8 (14.1)	n.s.
**Macronutrients intake**			
Total energy [kcal/day]	2 100 (683)	2 072 (706)	n.s.
Proteins [g/day]	81 (29)	69 (38)	0.020
Lipids [g/day]	83 (49)	70.0 (35)	0.030
Carbohydrates [g/day]	232 (98)	250 (105)	0.030
Dietary fiber [g/day]	18 (10)	33 (20)	<0.001
**Glucose metabolism**			
Fasting glucose [mmol/l]	4.8 (0.3)	4.7 (0.4)	n.s.
2h OGTT glucose [mmol/l]	5.9 (1.4)	5.5 (1.3)	0.070
AUC for OGTT glucose			
[mmol/l x 120min^−1^]	255 (137)	184 (159)	n.s.
AUC for OGTT insulin			
[mIU/l x 120min^−1^]	4,416 (1938)	3,143 (2603)	0.004
Insulin [mIU/l]	3.9 (2.7)	3.4 (1.7)	n.s.
C-peptide [pmol/l]	232 (103)	229 (79)	n.s.
HbA1c [mmol/mol]	32.0 (2.5)	30.0 (4.0)	0.010
Matsuda index	10.2 (6.6)	9.9 (5.2)	n.s.
**Lipid metabolism**			
Total cholesterol [mmol/l]	4.3 (1.1)	3.3 (0.8)	<0.001
HDL-C [mmol/l]	1.7 (0.7)	1.4 (0.4)	<0.001
LDL-C [mmol/l]	2.4 (1.2)	1.7 (0.8)	<0.001
Triacylglycerols [mmol/l]	0.7 (0.5)	0.7 (0.4)	n.s.
**Inflammatory markers**			
CRP (mg/l)	0.074 (0.081)	0.045 (0.028)	<0.001
**Stool characteristics**			
pH in feces	7.3 (0.7)	6.9 (0.8)	0.005
dry mass (%)	25.1 (9.9)	20.3 (8.8)	0.002

*Data are given as median (interquartile range). BMI, body mass index; WHR, waist-hip ratio; FFM, fat-free mass; TBW, total body water; OGTT, oral glucose tolerance test; AUC, area under the curve during oral glucose tolerance test; CRP, C-reactive protein; HbA1c, glycated hemoglobin; HDL-C, high-density lipoprotein–cholesterol; LDL-C, low-density lipoprotein–cholesterol*.

### Fecal Microbiome Composition

**Figure 1:**
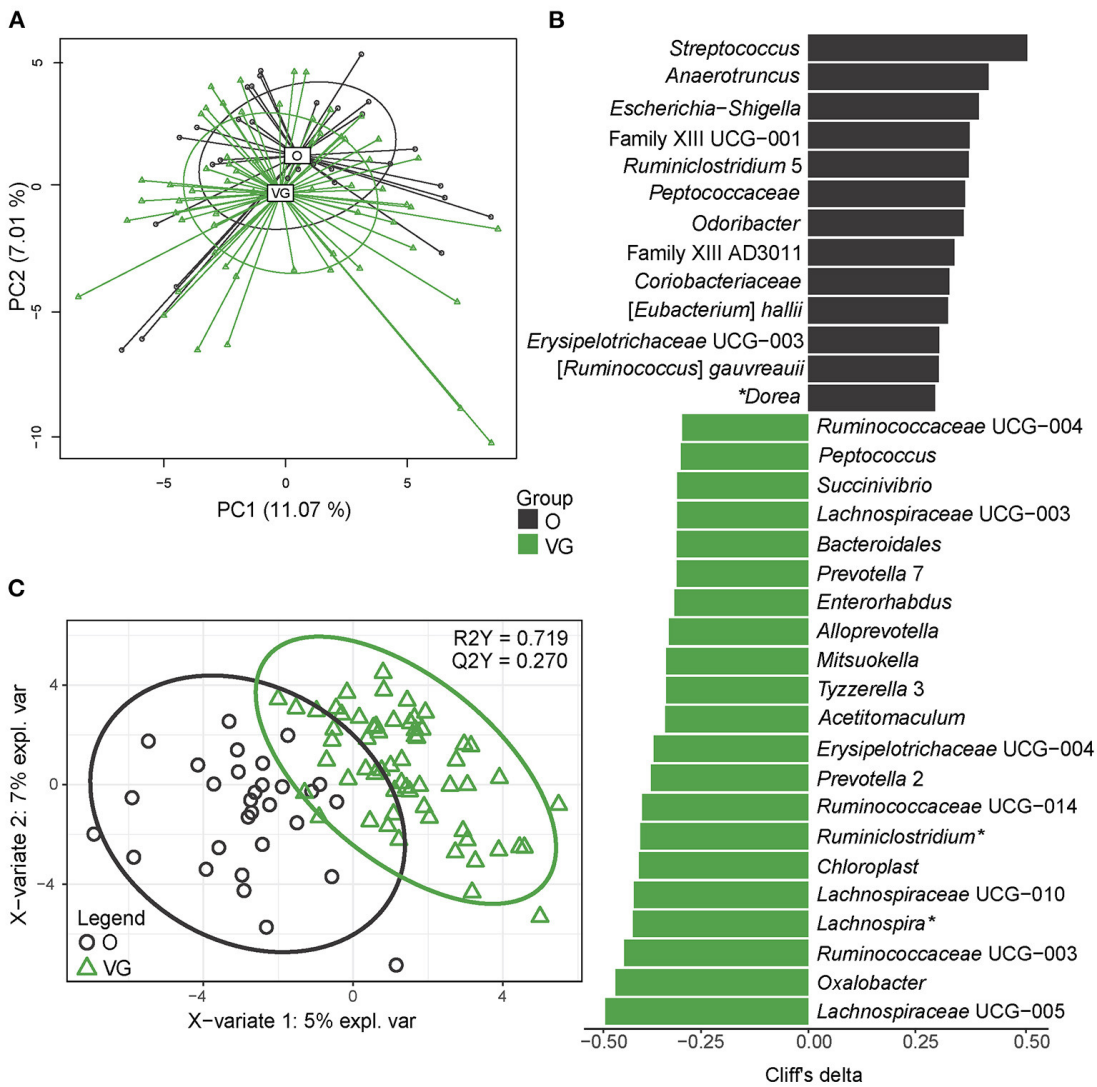
Gut microbiome composition. **(A)** Two-dimensional (2D) principal component analysis (PCA) score plot with the explained variance of each component. **(B)** Biomarker taxa generated from univariable discrimination analysis (FDR ≥ 0.1), effect size estimated by Cliff's delta; **(C)** 2D score plots of PLS-DA. R2Y fit goodness, Q2Y predictive power. *genera belonging to the core microbiome (abundance > 0.1%, prevalence > 75%). Effect sizes, FDR values, and variable importance in projection (VIP) values can be found in Supplementary Table 3. Data are presented as compositional (proportion of the particular bacteria of total sum of bacteria) after clr transformation.

Ninety fecal samples were available for microbiome analysis (57 from Os; 33 from VGs). In all the 90 samples, we identified 62,683 amplicon sequence variants (ASVs). Median sequencing coverage was 22,957 ASVs per sample (min 7,385; max 38,528). We detected 10 phyla, 19 classes, 24 orders, 44 families, and 144 genera; 55 of the genera belong to the core microbiome. For the purpose of this study, the core microbiome was defined as taxa (genus level) that meet the following criteria: median abundance of 0.1% and prevalence >75% at least in one group. The normalized α-diversity of the gut microbiota was estimated using indexes measuring richness (observed species) and evenness (Chao1, Shannon, Simpson, Pielou). In all the parameters, diversity was higher in the omnivore group (Supplementary Table 4). We identified 10 phyla dominated by *Firmicutes* (median abundance 46 and 52% in the vegans and omnivores, respectively) and *Bacteroidetes* (median abundance 44 and 41%) followed by much less abundant *Proteobacteria* (median abundance 1.6 and 1.6%)*, Actinobacteria* (median abundance 1.8 and 4.2%), and *Verrucomicrobia* (median abundance 0.2 and 0.4%). The abundance of other phyla was below 0.01%. Multivariable statistics (PERMANOVA) revealed significant differences in β-diversity at the level of order (*p* = 0.023), family (*p* = 0.013), and genus (*p* > 0.001) between the groups. The separation of vegans and omnivores at the genus level is visualized in Figures 1A,C using unsupervised (PCA) and supervised (PLS-DA) methods, respectively. Univariable differential abundance analysis followed by effect size analysis (Cliff's delta) identified 34 genera with significantly different abundance between the groups (FDR ≥ 0.1) (Figure 1B, Supplementary Table 5). As next step, we employed a PLS-DA model in order to address mutual relationships among the variables. According to this model, characterized by R2Y = 0.719 (goodness of fit) and Q2Y = 0.27 (goodness of prediction) metrics, we selected 55 genera with VIP value >1 (Supplementary Table 5). The combined set of variables selected by both approaches comprised 58 genera, representing 14.8% of total bacteria detected in both the vegans and omnivores. Ten of them belong to the core microbiome; three being enriched (*Lachnospira, Lachnospiraceae NK4A136* group, and *Ruminiclostridium*) and seven (*Alistipes, Bifidobacterium, Blautia, Fusicatenibacter, Dorea, Anaerostipes*, and *Ruminococcaceae_uncultured)* being depleted in the vegans compared with the omnivores. The remaining genera belong to the low abundant (0.1%) and very low abundant (0.01%) and rare taxa; the former being enriched rather in the omnivores, while the latter mostly in the vegans. To explore whether we could discriminate between vegans and omnivores according to microbiome composition, we employed a machine learning approach, specifically, a random forest algorithm. As we had only 90 subjects, we adopted 10-fold cross-validation to avoid reporting insignificant results for an overfitted model (the same method was used for the calculation of Q2Y metrics for PLS-DA). For fecal microbiome, we reached 83% accuracy of discrimination between vegans and omnivores, *p* < 0.01, obtained by permutation test. Nevertheless, this model tended to misclassify the vegans as controls with a false positive rate of 36.7%.

### Functional Capacity of the Gut Microbiota: But Gene Abundance

Having in mind the limitations of 16S rRNA gene sequencing regarding resolution power, we tried to characterize gut microbiota independently on taxonomic classification by searching for markers of its functional capacity. We determined the abundance of butyryl-CoA:acetate CoA-transferase (*but*) gene, encoding the key enzyme of butyrate synthesis. We employed the qPCR method based on degenerate primers that allow for covering a wide spectrum of *but* gene variants. As shown in Table 2, we did not identify any difference in *but* gene abundance in gut microbiome in the vegan and omnivore groups.

**Table 2:** Normalized *but* gene copy number.

**Cluster**	**Copy number**		***p*-value**	**FDR**	**Cliff's delta**
	**Omnivore**	**Vegan**			
A	4.0 (2.3)	3.5 (2.0)	0.056	0.337	−0.3
B	0.20 (2.25)	0.44 (5.09)	0.135	0.404	−0.2
C	190 (310)	211 (243)	0.289	0.432	−0.1
D	31 (111)	63 (118)	0.360	0.432	0.1
E	0.48 (0.55)	0.28 (0.48)	0.269	0.432	0.1
F	22 (40)	12 (25)	0.647	0.647	0.1

*Data are given as median (interquartile range). The copy number of the but gene was normalized to spike DNA (C. elegans UNC-6 gene) and calculated using the ΔCt method. Clusters represent groups of bacteria sharing sufficient but gene similarity allowing for the use of one degenerate primer pair. Bacteria belonging to individual clusters are listed in Supplementary Table 9*.

### Fecal Metabolome

**Figure 2:**
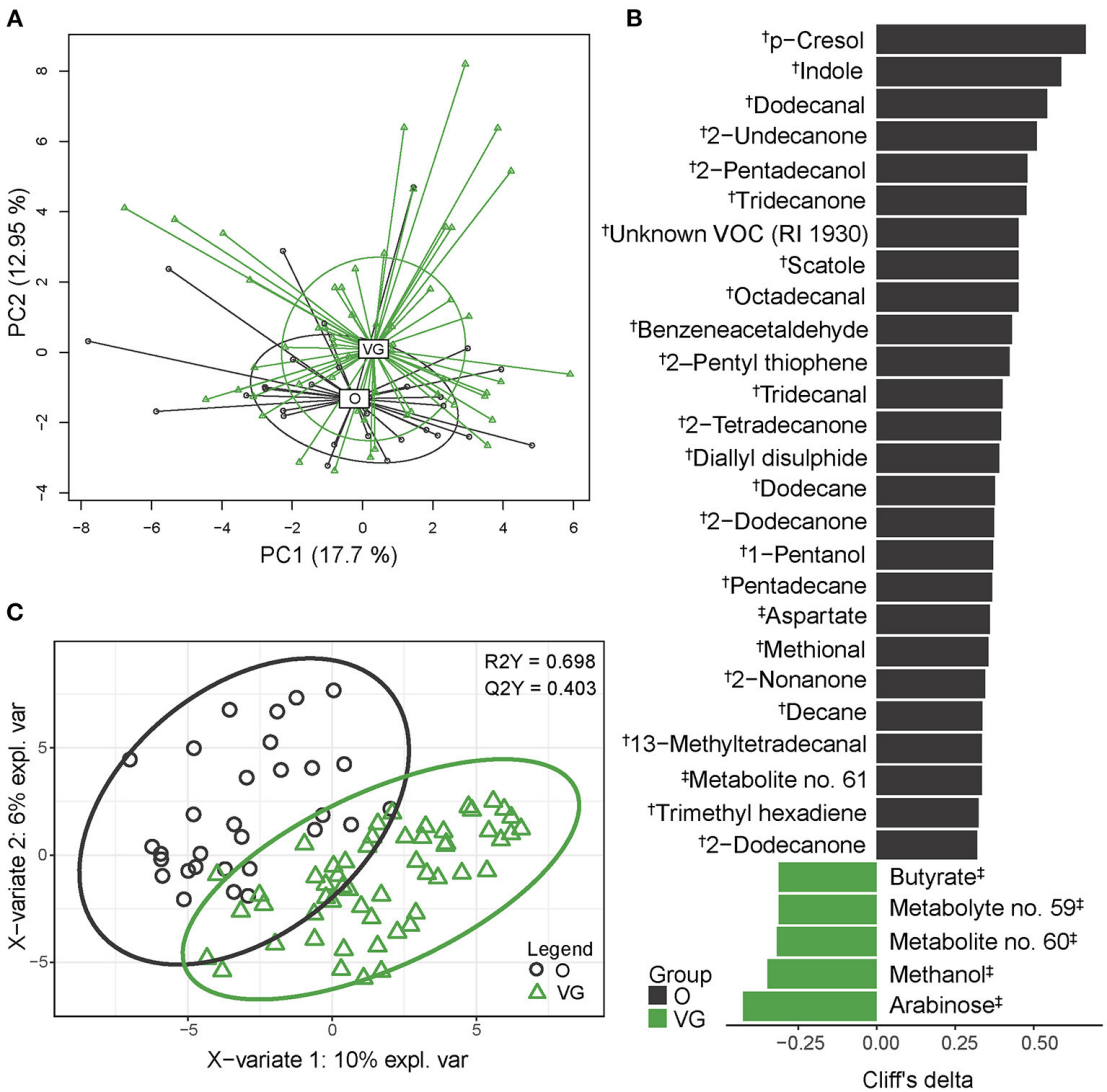
Fecal metabolome composition. **(A)** 2D PCA analysis score plot with the explained variance of each component. **(B)** Biomarker metabolites generated from univariable discrimination analysis (FDR ≥0.1), effect size estimated by Cliff's delta. **(C)** 2D score plots of PLS-DA. R2Y fit goodness, Q2Y predictive power. ^†^metabolites identified by GC-MS; ^‡^metabolites identified by NMR. Effect sizes, FDR values and VIP values can be found in Supplementary Table 4. Volatile compound (VOC) abundances are presented as compositional after clr transformation, NMR data were normalized by probabilistic quotient normalization (PQN).

We analyzed the fecal metabolome using two approaches, each of them covering a different spectrum of metabolites. By SPME-GC-TOF-MS, we identified 146 different VOCs, of which 80 were very low abundant (>0.1%), 52 were low abundant (0.1–1%), 10 were medium abundant (1–5%), and 4 (p-cresol, indole, scatole, ethyl butyrate) were highly abundant compounds (>5 %). The NMR spectrum comprised 34 quantified analytes. Only two compounds, i.e., butyric acid/butyrate and valeric acid/valerate, were identified by both approaches, and in both cases the values obtained by different methods correlated. For further statistical analysis, both datasets were combined and analyzed together. The separation of vegans and omnivores is visualized in Figure 2A. Multivariable statistics (PERMANOVA) revealed significant differences in β-diversity (*p* = 0.0045) between the groups. Univariable analysis revealed that abundances of 32 analytes differed significantly between the vegan and the omnivore groups (Figure 2B), and that the PLS-DA model (R2Y = 0.698; Q2Y = 0.403) selected 70 compounds (Figure 2C, Supplementary Table 6). The set of variables identified by both approaches comprised 77 compounds, representing 43.3% of all the fecal metabolites detected in both the vegans and omnivores. Vegan fecal metabolome was enriched by products of polysaccharide fermentation, i.e., short-chain fatty acids (SCFAs), such as butyrate and acetate and their derivatives (*n* = 18), medium-chain fatty acids (MCFAs) and their derivatives (*n* = 5), and further by methanol, monosaccharides, and several other compounds. In contrast, amino acid fermentation products, such as three most abundant metabolites (p-cresol, indole, and scatole) were approximately 50% lower in the vegans. The vegan fecal metabolome was also characterized by lower content of aromatic compounds benzacetaldehyde or 2-pentyl thiophene, medium- or long-chain alcohols, ketones, and aldehydes. The classification accuracy of the random forest model reached 76.2% (*p* < 0.01, obtained by permutation test). The false positive rate was 43.3%.

### Bile Acid Profile in Feces

By targeted LCQ-TOF-MS, we identified 11 bile acids. PERMANOVA analysis did not provide significant differences between the vegans and omnivores. Univariable analysis revealed significantly lower lithocholic acid (LCA) content in the samples of vegans. LCA was also the most abundant bile acid (Supplementary Table 7).

### Serum/Plasma Metabolome

**Figure 3:**
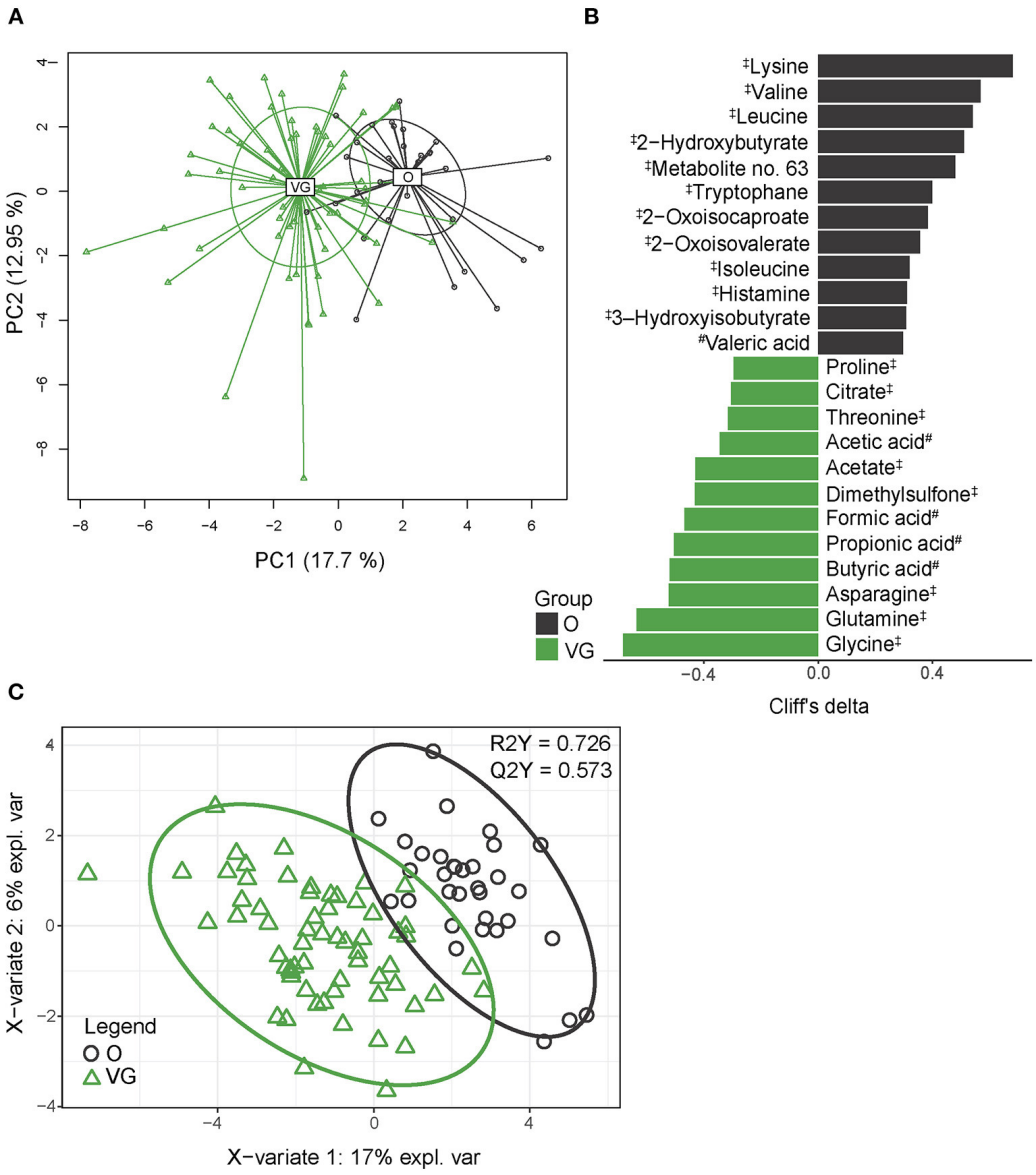
Serum metabolome composition. **(A)** 2D PCA analysis score plot with the explained variance of each component. **(B)** Biomarker metabolites generated from univariable discrimination analysis (FDR ≥ 0.1), effect size estimated by Cliff's delta. **(C)** 2D score plots of PLS-DA. R2Y fit goodness, Q2Y predictive power. ^#^metabolites identified by LC-MS; ^‡^metabolites identified by NMR. Effect sizes, FDR values, and VIP values can be found in Supplementary Table 6. NMR data were normalized by PQN.

To identify the composition of serum metabolome, we employed an untargeted NMR approach and LC-MS analysis allowing for the exact determination of SCFA concentration in the plasma. Altogether, we identified 34 quantified analytes by NMR and nine SCFAs by LC-MS, and only acetate/acetic acid was identified by both methods. For further statistical analysis, both datasets were combined and analyzed together. PCA shows clear separation of vegan and omnivore serum metabolome (Figure 3A), which was confirmed by PERMANOVA (*p* > 0.001). Univariable analysis identified 24 metabolites (of which 15 were amino acids or their derivatives and five were SCFAs) significantly differentially abundant between the groups (Figure 3B). The PLS-DA model (R2Y = 0.726, Q2Y = 0.573) selected 15 metabolites with VIP > 1 (Figure 3C, Supplementary Table 8). The set of variables selected by both approaches comprised 24 compounds, representing 55.8% of all the detected serum metabolites. Vegan serum metabolome is characterized by higher content of SCFAs (formic, acetic, propionic, and butyric acids), dimethylsulfone, and amino acids glycine, glutamine, asparagine, proline, and threonine, while the concentrations of branched-chain amino acids (BCAAs), their derivatives, and essential amino acid lysine were lower. Some of these metabolites, i.e., SCFAs, dimethylsulfone, and BCAA derivatives are potential co-metabolites of host and bacterial metabolism. The classification accuracy of the random forest model built on serum metabolome data reached 91.6% (*p* < 0.001, obtained by permutation test); false positive rate was 18.2%.

### Urine Metabolome

**Figure 4:**
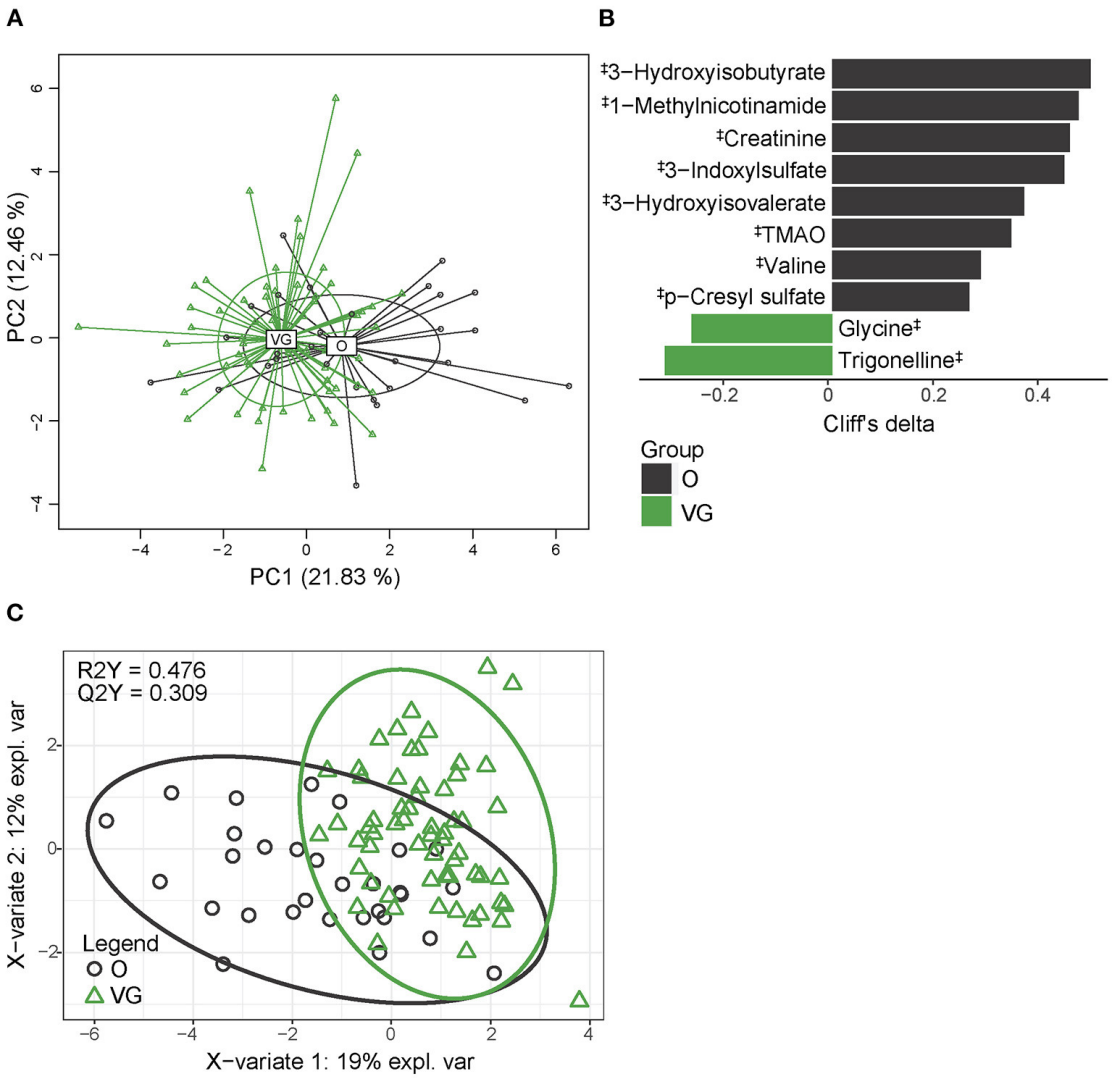
Urine metabolome composition. **(A)** 2D PCA analysis score plot with the explained variance of each component; **(B)** Biomarker metabolites generated from univariable discrimination analysis (FDR ≥ 0.1), effect size estimated by Cliff's delta. **(C)** 2D score plots of PLS-DA. R2Y fit goodness, Q2Y predictive power. ^‡^metabolites identified by NMR. Effect sizes, FDR values, and VIP values can be found in Supplementary Table 7. NMR data were normalized by PQN.

Urine metabolome, determined by untargeted NMR analysis, comprised 18 quantified metabolites and significantly differed between the groups (PERMANOVA, *p* > 0.001). The distribution of vegans and omnivores is shown in Figure 4A. Univariable analysis identified 10 metabolites that differed in abundance in the vegan and omnivore groups (Figure 4B), while the PLS-DA model (R2Y = 0.476, Q2Y = 0.309) selected seven discriminatory compounds (Figure 4C, Supplementary Table 9). The list of variables selected by both approaches comprised 12 compounds, representing 66.7% of all the detected metabolites. Ten of these compounds were depleted in the vegan urine metabolome, and all of them were related to protein/amino acid metabolism. Only glycine and trigonelline were higher in the vegans. The discrimination power of the random forest algorithm was 78.3% (*p* < 0.01, obtained by permutation test); false positive rate was 46.7%.

### Network Analysis

**Figure 5:**
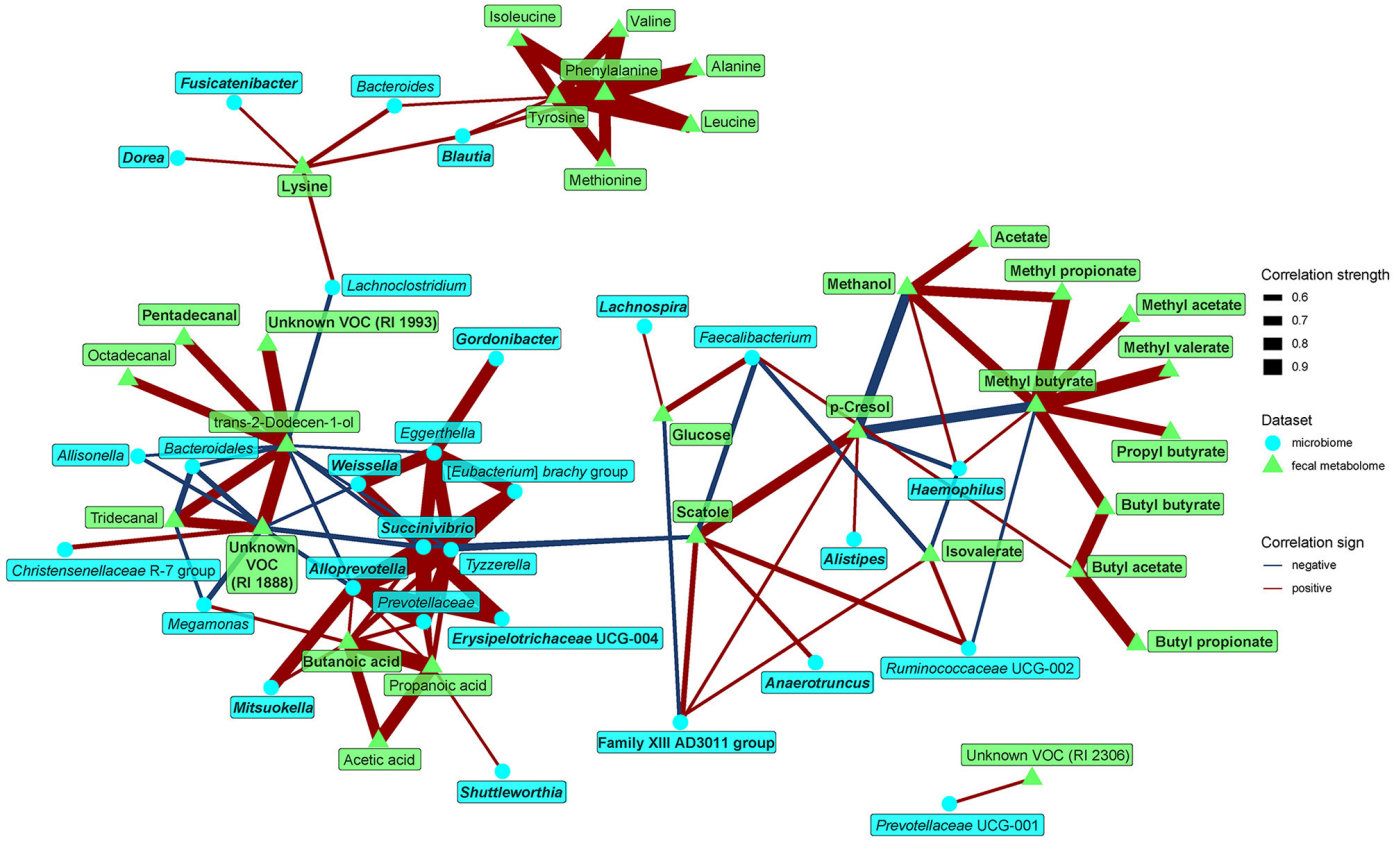
Spearman's correlations between bacteria and fecal metabolome components. The compositional data (values of bacteria and VOC abundance) were clr transformed prior to the construction of the networks, whereas data obtained from NMR were normalized by PQN. The edge width and color are proportional to the value of the correlation (red: positive; blue: negative). The features highlighted in bold were selected as significantly contributing to the discrimination between groups.

Finally, we looked for possible relationships between microbiome composition and metabolomic biomarkers, pooling data from both groups to gain contrast and power. Looking at the relationships between fecal microbiome and metabolome (Figure 5), we identified several motifs. First, the dominant tyrosine metabolites p-cresol and scatole positively correlated with *Anaerotruncus, Alistipes, Family XIII* AD3011 group, and *Ruminococcaceae* UCG-002. These metabolites further negatively correlated with methanol and several SCFA esters. Second, amino acids, such as BCAAs, lysine, tyrosine, phenylalanine, and methionine, positively correlated with *Bactreoides, Blautia, Dorea, Lachnoclostridium*, and *Fusicatenibacter*. With the exception of *Bacteroides*, all these bacteria were enriched in the omnivores. Third, a cluster of rare genera more represented in the vegan microbiome (*Tyzzerella, Succinivibrio, Shuttleworthia*, etc.) positively correlated with SCFAs (acetic, propionic, and butyric acids).

**Figure 6:**
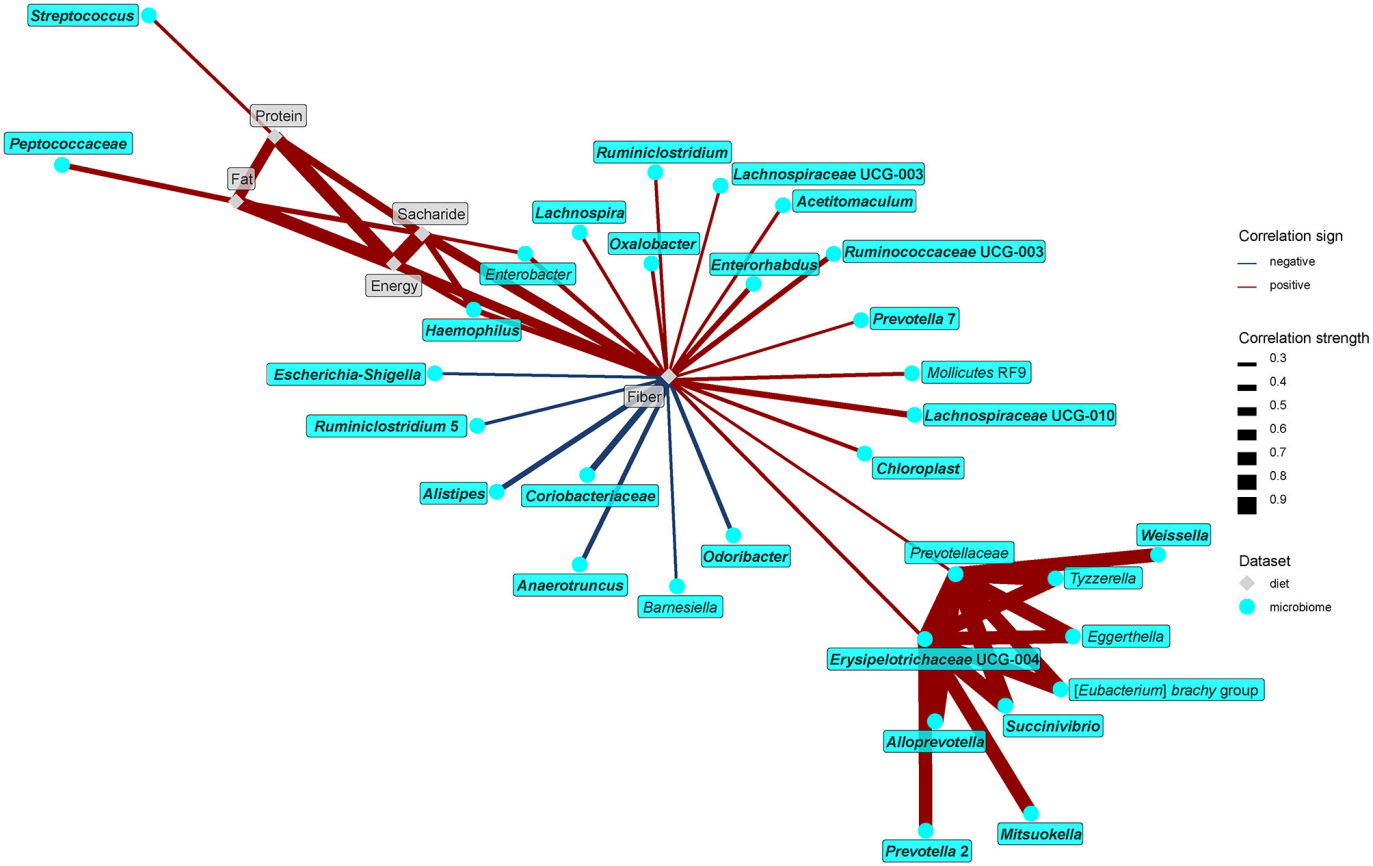
Spearman's correlations between bacteria and dietary components. The compositional data (values of bacteria abundance) were clr transformed prior to the construction of the networks. The edge width and color are proportional to the value of the correlation (red: positive; blue: negative). The features highlighted in bold were selected as significantly contributing to the discrimination between groups.

Examination of the relationships between fecal bile acids and microbiota identified LCA as the dominant compound at the interface of both datasets. LCA concentration in feces positively correlated with the abundance of *Ruminococcaceae* UCG-002 and *Family XIII* AD3011 group, both of them also correlated positively with protein fermentation products. In contrast, LCA negatively correlated with the abundance of nine bacteria enriched in the vegans, with two of them (*Lachnospira, Ruminiclostridium*) belonging to the core microbiota (Figure 6).

**Figure 7:**
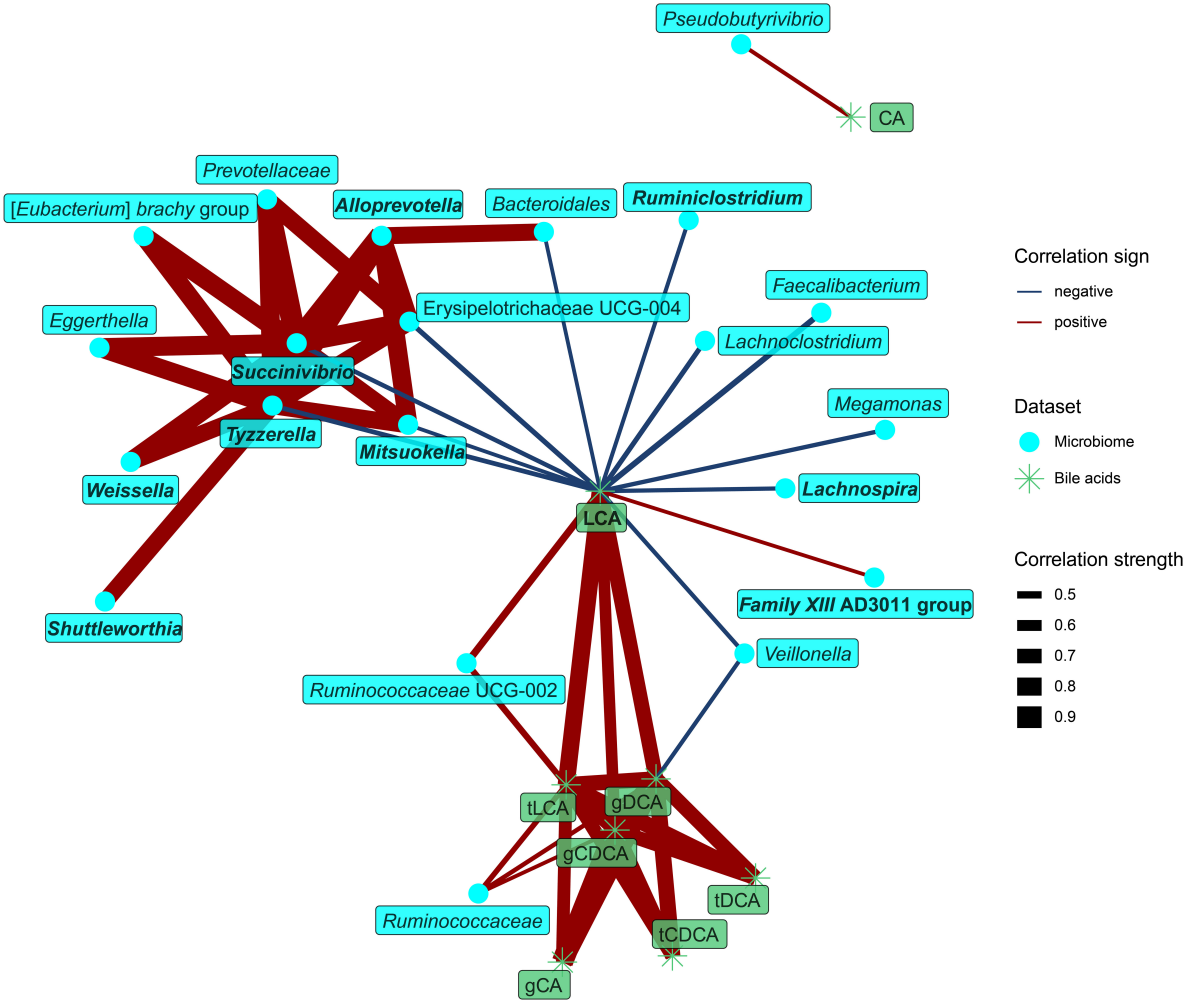
Spearman's correlations between bacteria and fecal bile acids. The compositional data (values of bacteria abundance) were clr transformed prior to the construction of the networks. The edge width and color are proportional to the value of the correlation (red: positive; blue: negative). The features highlighted in bold were selected as significantly contributing to the discrimination between groups.

**Figure 8:**
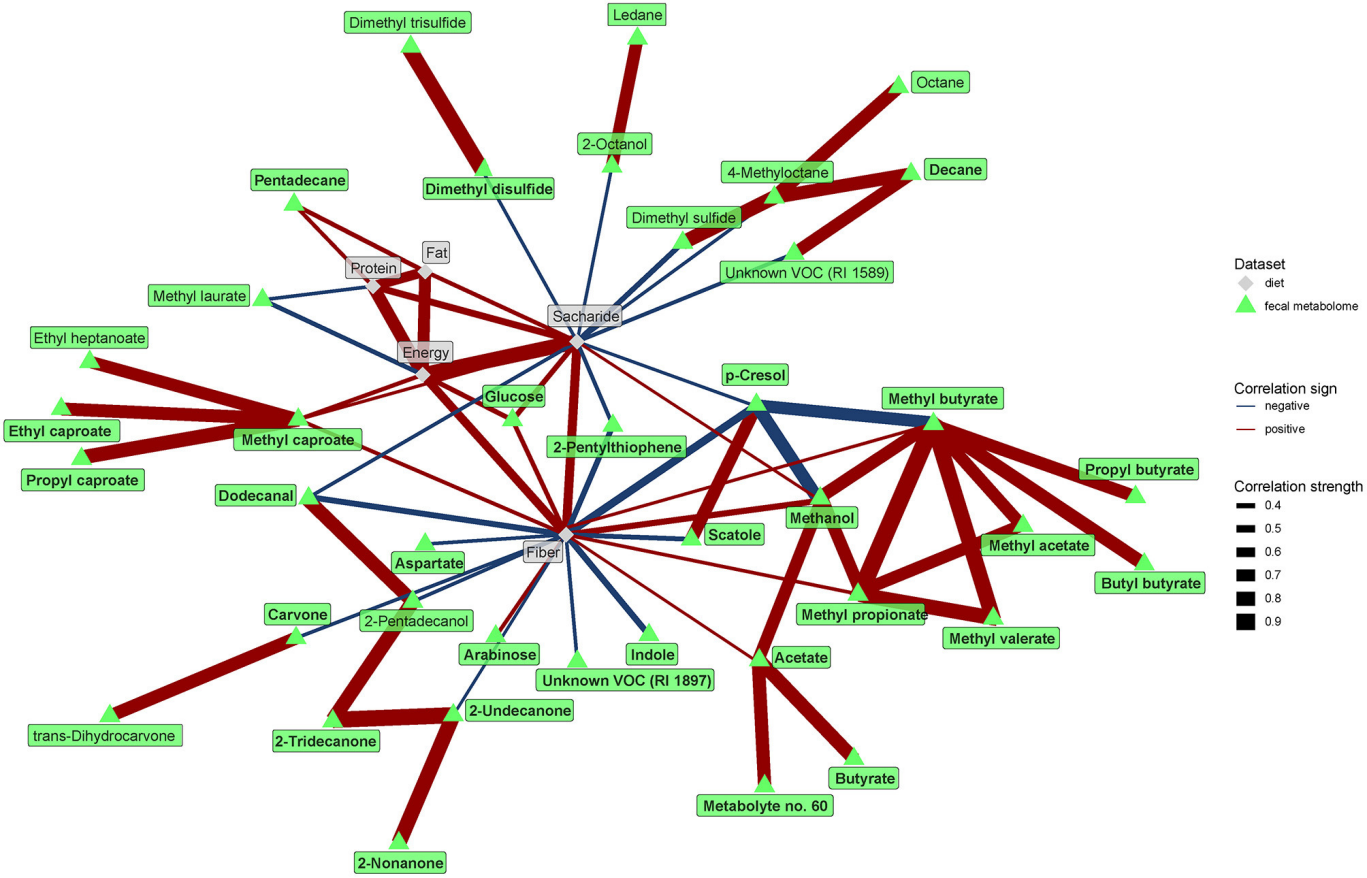
Spearman's correlations between fecal metabolome and dietary components. The compositional data (values of VOC abundance) were clr transformed prior to the construction of the networks. The edge width and color are proportional to the value of the correlation (red: positive; blue: negative). The features highlighted in bold were selected as significantly contributing to the discrimination between groups.

The network analysis further unraveled the central role of dietary fiber in the modulation of gut microbiome and metabolome. As expected, fiber positively correlated with many bacteria enriched in the vegan microbiome and negatively with some of those typical for the omnivores (Figure 7). Interestingly, negative associations with omnivore-characteristic bacteria were less frequent than positive associations with vegan-characteristic bacteria. Dietary protein correlated positively with *Streptococcus* and dietary fat with *Peptococcaceae*. Dietary fiber correlated negatively with numerous products of protein fermentation, and positively with methanol, acetate, butyrate, and SCFA esters (Figure 8).

**Figure 9:**
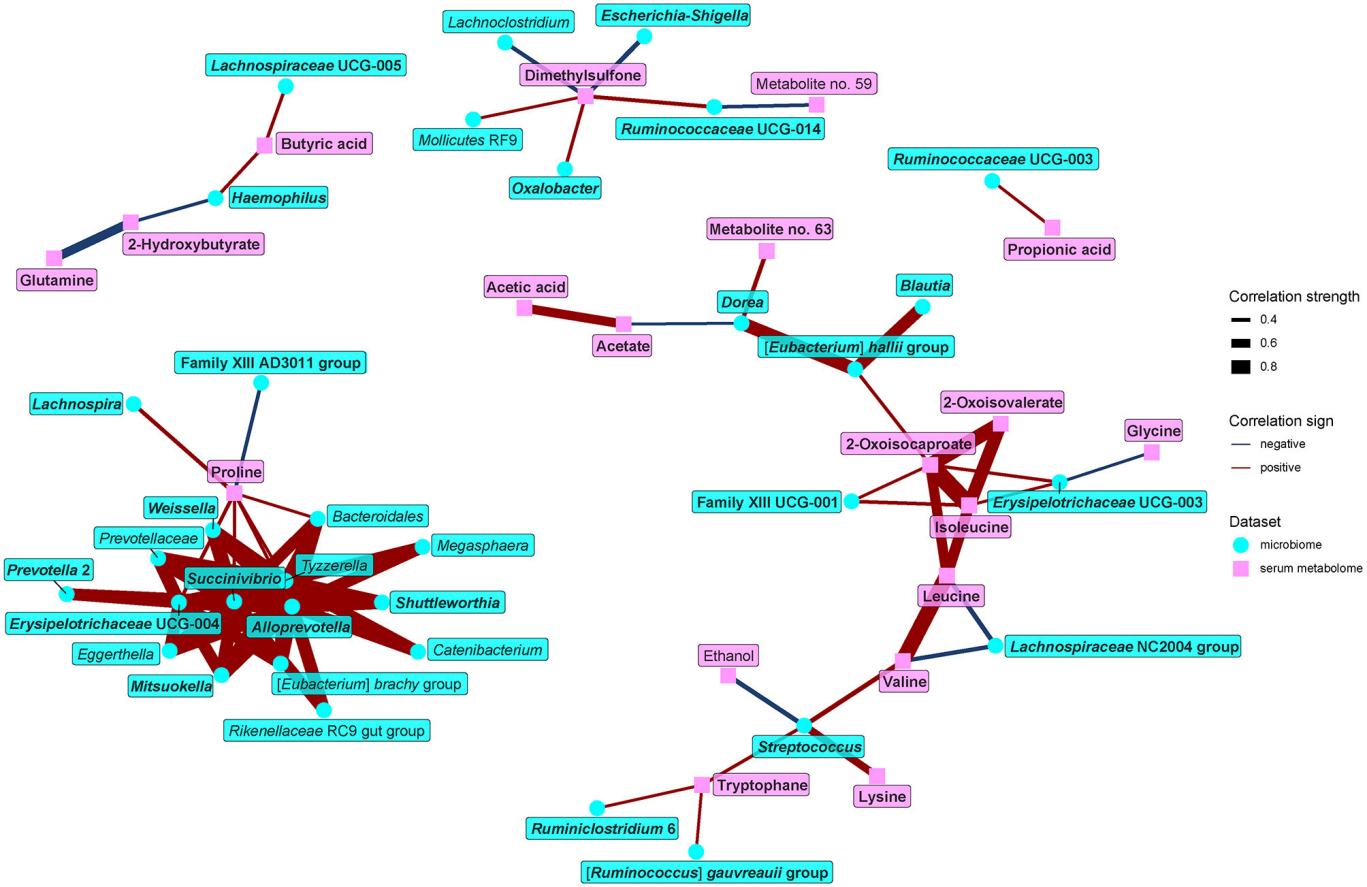
Spearman's correlations between bacteria and serum metabolome components. The compositional data (values of bacteria abundance) were clr transformed prior to the construction of the networks, whereas the data obtained from NMR were normalized by PQN. The edge width and color are proportional to the value of the correlation (red: positive; blue: negative). The features highlighted in bold were selected as significantly contributing to the discrimination between groups.

Because some of the serum metabolites contributing to the separation of vegans and omnivores may be of microbial origin, we further looked for associations between gut microbiome and serum metabolome (Figure 9). The main findings may be summarized as follows: first, BCAAs and their derivatives positively correlated with bacteria enriched in the omnivore microbiome (*Family XIII* UCG-001, *Erysipelotrichaceae* UCG-003, *Streptococcus, Eubacterium hallii* group*, Dorea*, and *Blautia*) and negatively with vegan-characteristic bacteria *Lachnospiraceae* NC2004 group. Second, tryptophan positively correlated with *Streptococcus, Ruminclostridium 6*, and the *Ruminococcus gauvreauii* group, all of which were higher in the omnivores. Third, proline positively correlated with numerous vegan-characteristic microbes and negatively with the *Family XIII* AD3001 group, omnivore-characteristic bacteria. Fourth, we found positive correlations between serum SCFAs and some of the vegan-characteristic bacteria, particularly propionate/*Ruminococcaceae* UCG-003 and butyrate/*Haemophilus*, and *Lachnospiraceae* UCG-005. *Dorea* (omnivore-characteristic bacteria) negatively correlated with acetate. Finally, dimethylsulfone negatively correlated with omnivore-characteristic genera (*Escherichia-Shigella*, and *Lachnoclostridium*) and positively with vegan-characteristic bacteria *Ruminococcaceae* UCG-014 and *Oxalobacter*.

Taken together, our findings indicate not only relationships among diet, fecal metabolome, and fecal microbiome but also more far-reached links connecting gut microbiota with metabolites in the serum.

## Discussion

To fill knowledge gaps in mechanisms linking the effects of diet and intestinal microbiota, we explored differences in the intestinal microbiota and related metabolites in a model population of vegans vs. omnivores. The groups clearly differ in their eating habits. The major finding of this study is that dietary composition relates to distinct gut microbiota metabolic performance and metabolomic features despite highly similar established proxies of metabolic health across the groups. Our finding of slightly greater alpha-diversity of gut microbiota among the non-vegans was unexpected but may be related to the fact that the control group consisted of younger volunteers with a rather favorable pattern of lifestyle factors.

### Vegan Diet and Microbiome Composition

The vegan diet is characterized by a different nutrient composition when compared to the omnivore diet, i.e., lower amounts of fat and protein, different patterns of amino acids, and higher amounts of dietary fiber. All of these features have a potential to promote important alterations of the gut microbiota (20) but available studies, recently reviewed in detail by Trefflich (21) and Losno (22), reported variable outcomes regarding changes in the overall composition of the microbiome associated with adherence to a vegan diet. The most reported groups affected by the vegan diet were Bacteroidetes and Firmicutes at the phylum level and *Bacteroides, Prevotella*, and *Bifidobacterium* at the genus level (22). In our study, we found only modest differences in microbiome composition associated with a vegan vs. omnivore diet, as only 14.8% of all the identified bacteria were affected by the diet, and the machine-learning algorithm based on microbiome data was quite inefficient in discriminating between vegans and omnivores. Nevertheless, it is important to mention that our results, as well as majority of other studies focused on vegan microbiota, are based on 16S rRNA hypervariable amplicon sequencing. Compared to shotgun sequencing, this method does not allow for more detailed taxonomy classification at the level of species and strain. We definitely cannot exclude the possibility that there are substantial differences between both groups at these levels. De Filippis et al. (23) demonstrated that different oligotypes within the same genus showed distinctive correlation patterns with dietary components and metabolome. *Prevotella* is a typical representative of plant-based diet-associated microorganism, while *Bactreoides* is linked to animal-based nutrients. Nevertheless, within both genera, oligotypes exist that associate with the opposite dietary component than the majority. Therefore, the diet/microbiome associations based only on genus-level taxonomy may be oversimplified and may not catch more subtle relationships.

On the other hand, taxonomic assignment only has limited ability to describe the functional capacity of the microbiota community. In addition to the non-optimal resolution of 16S rRNA sequencing, even well-assigned bacteria may possess unexpected characteristics because of the horizontal gene transfer that readily occurs among bacteria. Shotgun sequencing is the gold-standard method for the identification of full set of genes present in the bacterial community, but it is also costly and demanding for bioinformatics capacity. We developed an alternative approach based on qPCR quantification of gene of interest in stool DNA. In this study, we quantified the abundance of the *but* gene coding a key enzyme of butyrate synthesis. We chose this gene, as butyrate synthesis is one of the potential final steps of fiber fermentation, and butyrate is an important fermentation product with a significant impact on host health. We speculated that it could be a good readout of the effect of a diet rich or poor in fiber on microbiota composition. Somewhat surprisingly, we did not prove a significant difference in *but* abundance in the vegan and omnivore groups. This finding supports the results of taxonomic analysis.

Alpha diversity was higher in the omnivores than in the vegans, which is in disagreement with previously published results that showed no difference (24, 25) or higher diversity in vegans (26) but in line with a recent report comparing vegan and omnivore cohorts in Germany (27). The lack of significant effects of a long-term vegan diet or diet with high amounts of dietary fiber from cereals on gut microbiome composition observed by us and others (25, 28) is in contrast with significant differences that have been demonstrated between people from traditional agrarian societies dependent on mostly plant-based diets and Westernized societies consuming low-fiber high-protein diets (29, 30). Nevertheless, this observation still does not contradict the profound effect of diet on microbiota composition. Evaluation of archaic native American coprolith remains suggests that pre-agricultural fiber intake exceeded 100 g/day (31). This corresponds to estimated daily intake of fiber in surviving traditional societies still sticking to hunter-gatherer way of life, which is 80–150g/day (32, 33). In contrast to it, the median fiber intake in our vegan cohort was 33 g/day, and the value 100 g/day was exceptional. Furthermore, recent research indicates that microbiome composition is established in early childhood (34) and is relatively stable during adulthood. None of the vegans included into our study has been vegan since childhood, so the formation period of her/his microbiota occurred in omnivore setting. Taking into account all these circumstances, our findings support the hypothesis that the core gut microbiota (abundance > 0.1%, prevalence > 75%), at least at the genus level, are stable and resilient to compositional change, even during a long-term dietary shift in adulthood. However, we cannot rule out that compositional differences between the vegans and non-vegans would become apparent at the metagenomics level.

### Vegan Diet and Fecal Metabolome

Although DNA-based fingerprint procedures provide information about the composition of the microbial community, they do not reflect the metabolic activity of the populations (35). In contrast, fecal metabolome has been proposed as a functional readout of the human microbiome (36), reflective of microbiome–host interactions with immediate impact on host health. Bacterial genome often encodes genes for alternative metabolic pathways allowing for high metabolic flexibility. Bacteria are able to switch among different metabolic programs depending on available substrate in order to reach maximal energy extraction efficacy. The same bacteria are, therefore, capable to produce a very different spectrum of metabolites. Thus, we performed untargeted fecal metabolome analyses as a functional readout. Among the important characteristics of vegan fecal metabolome was significantly lower content of amino acid fermentation products p-cresol, indole, scatole, and some aromatic compounds that were consistently identified by two independent statistical methods. These metabolites also belong to the most abundant components of fecal metabolome. The second group of compounds discriminating between vegans and omnivores are SCFA and SCFA-derived esters. Most bacteria possess multiple metabolic programs that may be switched on and off according to the available substrate and environmental conditions. The composition of the fecal metabolome, therefore, reflects the preferential carbohydrate fermentation in vegans and shift to protein fermentation in omnivores because of the different diet macronutrient composition of their diets. The vegan diet contains less protein but more fiber than the omnivore one. Amino acids are less efficient as energy source for human gut microbes; therefore, gut microbiota preferentially consume carbohydrates over proteins (37). This shift in fecal metabolome composition has implications for human health, as products of protein fermentation, like p-cresol, have considerable health effects. It is associated with adverse effects such as genotoxicity, oxidative stress, compromised integrity of the gut epithelium, and decreased viability as well as proliferation of intestinal epithelial cells (37). Our study clearly demonstrated that a vegan dietary pattern is associated with distinct metabolomic profiles compared to a meat-containing diet, even when comparing vegan vs. non-vegan individuals with otherwise similar characteristics, e.g., regarding age and BMI. However, whether the observed differences underlie potential long-term health benefits of a vegan diet needs to be further investigated, for example, by comprehensive metabolomics assessments in epidemiological long-term studies.

### Gut Microbiota and Fecal Metabolome

Several potentially interesting associations between bacterial abundances and metabolites were revealed in this study. *Alistipes*, the *Family XIII*AD3011 group, and *Anaerotruncus*, all higher in omnivores, positively correlate with p-cresol and scatole. Three other bacteria significantly higher in omnivores (*Dorea, Blautia*, and *Fusicatenibacter*) positively correlate with amino acids tyrosine, BCAAs, methionine, lysine, phenylalanine, and valine. The association of these bacteria with metabolically adverse phenotypes was reported (38, 39). Not surprisingly, dietary fiber correlated (both positively and negatively) with most of bacteria differently represented in both groups. Accordingly, we found positive correlations between dietary fiber and SCFAs and their derivatives, as well as negative correlations between dietary fiber and protein fermentation products. Nevertheless, the fecal metabolome still did not discriminate well between both groups, probably because of factors such as metabolic cross-feeding between different bacterial groups, utilization of bacterial metabolites by the host, and technical issues concerning the difficult normalization of fecal metabolite concentration.

### Gut Microbiota and Fecal Bile Acids

Primary bile acids secreted from the liver to the intestine are subjects of extensive microbial transformation within the gut, and the spectrum of secondary bile acids reflects the composition and metabolic performance of gut microbiota. In our study, we did not observe any difference between the VG and O groups in the fecal concentration of primary bile acids and their derivatives but significantly lower concentration of one secondary bile acid, LCA, in VGs than in Os. Secondary bile acids are solely the product of microbial transformation of deconjugated primary bile acids (40); therefore, the significant difference in LCA concentration between vegans and omnivores with comparable primary BA synthesis may indicate different microbial activity. Recent research revealed the important role of bile acids as signals in the regulation of lipid and glucose metabolism (41) or in cancerogenesis (42). Type 2 diabetes is associated with higher plasma levels of LCA (43), and with significantly altered bile acid signature in feces (44). Our study only comprised metabolically healthy omnivores and vegans, so we cannot draw any strong conclusions regarding LCA fecal content and metabolic health, but even within physiological limits, the vegans had more favorable glucose metabolism-related parameters than the omnivores. Further research is needed to confirm or deny the hypothesis that microbial metabolism of bile acids contributes to the impairment of glucose homeostasis of a host.

### Gut-To-Circulation Crosstalk

The composition of serum/plasma metabolome is the most discriminative characteristic between vegans and omnivores. Our data indicate that it reflects both dietary pattern and gut microbiota activity. Without a doubt, the macronutrient, particularly protein intake and amino acid composition, is different in vegans and omnivores. The different diet composition has a direct effect on physiology, and absorption of nutrients in the upper gastrointestinal tract, therefore, had a limited impact on colonic microbiota. However, some of the metabolites differently abundant in vegan and omnivore serum are co-metabolites formed both by the host and gut microbiota advocating direct gut-to-circulation crosstalk. This is the case for dimethylsulfone, a product of microbial metabolism of methionine as well as BCAAs and SCFAs.

Reduced circulating BCAA in vegans has been observed previously (25, 45) and may relate to lower BCAA intake (46). Nevertheless, Wang et al. proved that the BCAA degradation pathway is upregulated in gut microbiota of vegans and vegetarians compared to those of omnivores (45). Therefore, upregulated BCAA degradation in the gut may contribute to the observed lower serum BCAA concentrations in vegans. The reduction of circulating BCAA may represent one of the microbiome-related mechanism contributing to the health-promoting effects of plant-based diet, as it has been repeatedly shown that increased circulating BCAA decline in metabolic health and diabetes development (47). We identified a less favorable metabolic phenotype of omnivores despite the fact that both groups in this study comprised volunteers with healthy normal weight and normal glucose tolerance.

Short-chained fatty acids (SCFAs) are products of bacterial fermentation of fiber in the gut, and while demonstrating their higher concentration in vegan fecal as well as serum metabolome we provide a direct link connecting microbiome activity in the gut and circulating metabolome.

### Strengths and Limitations

Our aim was to perform rigorous matching of participant characteristics across vegans and omnivores, and to focus on young and healthy population to minimize the risk of potential confounders influencing metabolic health. The main limitation of our study is that the results were obtained on rather small vegan and omnivore groups, and that the outcomes were not validated in an independent cohort. However, the results were internally validated through permutation tests. Thus, the main purpose of the methods employed was to demonstrate that certain domains of the metabolome provide better discrimination of vegans and omnivores, and possibly a better picture of differential functional consequences of the diets, compared to bacterial abundances alone. The outcomes deserve further validation in independent well-matched cohorts. Another potentially limiting aspect that must be taken into consideration is the methodology used for the characterization of microbiota composition. We based our analysis on the sequencing of the V4 region of 16S rRNA gene, which provides lower resolution than shotgun sequencing. This may lead to the underestimation of the effect imposed by vegan diet on gut microbiota composition especially at the sub-genus level. Finally, the diet of both groups was analyzed only at the macronutrient level, as the aim of this study was comparative analysis of microbiome composition and metabolomic footprints of vegans and non-vegans. While our analyses strongly suggest that omitting animal foods has a distinct effect on the metabolome, further studies are needed on the potential mediating role of nutrient intakes, which may underlie the observed metabolic differences between vegans and non-vegans, beyond macronutrient and fiber intake.

## Conclusion

We showed that the composition of gut microbiota of long-term vegans and omnivores is not dramatically different. In contrast, vegans and omnivores significantly differ in the composition of the fecal, serum, and urine metabolomes as an effect of different availability of substrates (dietary fiber vs. protein). Consequently, the vegan diet was associated with a lower abundance of the potentially harmful (protein fermentation products) and a higher occurrence of potentially beneficial (dietary fiber fermentation products) metabolites. While our study suggests that a shift toward a vegan diet may be an avenue to personalized manipulation of microbiome function, targeting metabolites with health implications such as indole or cresole (48), we acknowledge that our study was observational and that proof-of-concept RCTs are needed to investigate the potential of dietary interventions in this context. In general, while our study had the purpose of identifying metabolites that are differentially abundant between vegans and non-vegans, the clinical utility of the measured biomarkers for risk prediction or clinical monitoring is yet to be proven.

## Data Availability Statement

The datasets presented in this study can be found in online repositories. The names of the repository/repositories and accession number(s) can be found below: https://www.ebi.ac.uk/ena, PRJEB43938.

## Ethics Statement

The studies involving human participants were reviewed and approved by Multicentric Ethic Committee of Kralovske Vinohrady University Hospital. The patients/participants provided their written informed consent to participate in this study.

## Author Contributions

MP: resources, investigation, and writing of the original draft. EB and SS: methodology and formal analysis. MKu: investigation and writing-review and editing. HP, JH, PV, PS, and KK: investigation. MHec: investigation and project administration. ND: investigation and visualization. MB: data curation and project administration. IM: methodology, formal analysis, and visualization. MKr: methodology, formal analysis, and supervision. MHen and ES: resources and formal analysis. KC: investigation, supervision, and formal analysis. RS: writing-review and editing, and supervision. RL: methodology, investigation, and writing-review and editing. TK: methodology, supervision, and writing-review and editing. JG and MC: conceptualization, methodology, data curation, funding acquisition, and writing of the original draft. All authors contributed to the article and approved the submitted version.

## Funding

This study was supported by the Ministry of Health of the Czech Republic (Grant NV-18-01-00040), Charles University (Grant 1280218), institutional support PROGRES Q36, and MH CR -DRO Institute for Clinical and Experimental Medicine -IKEM (Grant IN00023001). The authors used services of the Czech Centre for Phenogenomics supported by the Czech Academy of Sciences RVO 68378050 and by the project LM2018126 Czech Centre for Phenogenomics provided by MYES CR.

## Conflict of Interest

The authors declare that the research was conducted in the absence of any commercial or financial relationships that could be construed as a potential conflict of interest.

## Publisher's Note

All claims expressed in this article are solely those of the authors and do not necessarily represent those of their affiliated organizations, or those of the publisher, the editors and the reviewers. Any product that may be evaluated in this article, or claim that may be made by its manufacturer, is not guaranteed or endorsed by the publisher.
