# Supplementary material for: Vegan Diet Is Associated With Favorable Effects on the Metabolic Performance of Intestinal Microbiota: A Cross-Sectional Multi-Omics Study
Source: Front Nutr. 2022 Jan 7;8:783302. doi: 10.3389/fnut.2021.783302 (PMC8777108; doi:10.3389/fnut.2021.783302)

# *Supplementary Material*

## 1 Supplementary Data

### Characterization of study cohorts

*Recruitment*

Between October 2018 and October 2019 the age, sex and BMI matched participants categorized as vegans (VG, n=62) or omnivore (O, n=33) were screened and enrolled. Vegans strictly avoided all animal products for at least three years, omnivore group comprised subjects without any dietary restrictions, who consumed meat and other animal products on a daily basis. In both groups, the exclusion criteria were age under 18 years, obesity, chronic diseases related to metabolism, diseases of the digestive tract, antibiotic therapy in the past three months, pregnancy, any chronic medication (excluding hormonal contraception) and regular alcohol consumption. All participants signed informed consent prior to enrollment. The research protocol was approved by the Ethics Committee of the 3$^{rd}$ Faculty of Medicine of Charles University and Ethics Committee of University Hospital Kralovske Vinohrady in accordance with the Declaration of Helsinki.

*Anthropometry and clinical examination*

Each subject underwent a basic medical check-up with an antropometric examination (height, weight, BMI, waist circumference and waist-to-hip ratio). Body composition was determined by bioimpedance analysis (Nutriguard-M, Data Input GmbH, Frankfurt, Germany). Resting metabolic rate was measured by indirect calorimetry and calculated by Harris-Benedict equation.

*Dietary assessment*

Each participant filled in a prospective questionnaire, where dietary data from three typical days were collected (two working days, one weekend day). NutriDan program was used for dietary intake calculations. Daily intake of carbohydrates, lipids, proteins and dietary fiber were calculated separately.

*Laboratory analysis*

Peripheral venous blood sample was drawn from each subject after 12 hours of fasting. Parameters of glucose homeostasis (fasting plasma glucose, glycated hemoglobin (HBA1c), C-peptide and insulin) and lipid profile (total cholesterol, high-density lipoprotein cholesterol, low-density lipoprotein cholesterol and triacylglycerides) were assessed in a certified hospital laboratory.  Serum zonulin was detected using Human Zonulin ELISA Kit (Elabscience).

*Insulin sensitivity and secretion*

Insulin sensitivity and secretion were evaluated using data from oral glucose tolerance test (OGGT). OGTT (75g glucose) was performed after 12-hour-long fasting according to WHO recommendation. First, baseline blood samples were drawn, than the sampling was done 30-minute intervals for two hours yielding 5 values for each subject. Incremental AUCs for glucose and insulin were calculated using trapezoid rule. Insulin sensitivity alone was expressed as Matsuda Index (MI) as published [5].

In detail. In AUC calculation, the fasting serum glucose level was subtracted from each value to adjust for variability in fasting serum glucose levels between subjects giving the incremental AUC. The formula used for calculation of AUC was:

$$AUC = 0 + 2 \times (G|30 - G_0) + 2 \times (G|60 - G_0) + 2 \times \frac{(G|90 - G_0) + (G_{120} - G_0)}{8} \times 120$$

where G with lower index number represents serum glucose (mmol/l) in the respective time during OGGT.

The formula used for calculation of MI was:

$$MI = \frac{10000}{\sqrt[2]{(I_0 \times G_0) \times (I_{mean} \times G_{mean})}}$$

where $I_0$ and $G_0$ stand for fasting serum insulin (mU/l) and glucose (mg/dl) and $I_{mean}$ a $G_{mean}$ stand for average serum insulin (mU/l) and glucose (mg/dl) during oGGT, which were counted as:

$$G_{mean} = \frac{G_0 + 2 \times G_{30} + 2 \times G_{60} + 2 \times G_{90} + G_{120}}{8}$$

$$I_{mean} = \frac{I_0 + 2 \times I_{30} + 2 \times I_{60} + 2 \times I_{90} + I_{120}}{8}$$

**Sample manipulation and storage**

Stool collected at home was immediately stored at −20°C until transported in the frozen state to the laboratory. Once thawed, the four fold of water was added to the sample (up to 10 g), and samples were homogenized using stomacher (BioPro, CR). Immediately after homogenization, an aliquot (600 ul) was taken for DNA analysis. pH was determined in the rest of the sample and the homogenate was sonicated for 1 minute at the maximal amplitude and cycles (UP200S, Heischler Ultrasound Technology). Sonicated samples were used for dry mass estimation and aliquoted and stored at -50°C until metabolome analysis. For determination of bile acid spectrum, the sample aliquot was lyophilized. Urine samples were taken in the hospital and immediately frozen at -20°C until analysis.

Blood samples were drawn from median cubital vein into Vacutainer tube. For serum, the blood was left standing on the bench for 30 min to clot and then separated by centrifugation. For plasma, the blood was collected into Vacutainer with the anticoagulant, immediately mixed by gently inverting the tube five times and then separated by centrifugation. Parameters of glucose homeostasis were measured in a certified hospital laboratory: fasting plasma glucose using the hexokinase reaction (KONELAB, Dreieich, Germany); C-peptide by using solid-phase competitive chemiluminescent enzyme immunoassay (Immulite 2000, Los Angeles, CA, USA); HbA1c by using high-pressure liquid boronate affinity chromatography (Primus Corporation, Kansas city, MO, USA); and insulin using solid-phase competitive chemiluminescent enzyme immunoassay (Immulite 2000). For the lipid profile, we measured total cholesterol and

triglycerides using an enzymatic method kit (KONELAB); high-density lipoprotein–cholesterol measured using a polyethylene glycol-modified enzymatic assay kit (ROCHE, Basel, Switzerland); and low-density lipoprotein–cholesterol calculated using the standard Friedewald equation.

**Gut microbiome analysis**

*Fecal Sample Collection and Bacterial DNA Extraction.*

Stool collected at home was immediately stored at -20°C until transported in the frozen state to the laboratory. Until isolation, samples were stored at -50°C. For DNA isolation, 200-250 mg of stool was cut on dry ice. DNA was isolated by QIAmp PowerFecal DNA Kit (Qiagen), according to manufacturer recommendation. Ninety samples (60 vegans, 30 omnivores) were analyzed.

*16S rRNA gene Library Preparation and Sequencing*

Quality of DNA was determined using gel electrophoresis and concentration was assessed spectrophotometrically using microplate reader (Synergy Mx, BioTek, USA). For identification of bacteria presented in samples, the sequencing of 16S rRNA gene was performed. Extracted DNA was used as a template in amplicon PCR to target the hypervariable region V4 of the bacterial 16S rRNA. The library was prepared according to the Illumina 16S Metagenomic sequencing Library Preparation protocol with some deviations described below (1). The total reaction volume of PCR was 30 μl consisting of 15 μl Q5 HighFidelity 2x MM (BioLabs, New England), 1.5 μl of each 10 μM primer, 9 μl of PCR water and and 3 μl of template. The cycling parameters included initial denaturation at 98°C for 30 s, followed by 30 cycles of 10 s denaturation at 98°C, 15 s annealing at 55°C and 30s extension at 72°C, followed by final extension at 72°C for 2 min. The primer pair consisting of Illumina overhang nucleotide sequences, an inner tag and gene-specific sequences. The Illumina overhang served to ligate the Illumina index and adapter. Each inner tag, i.e. a unique sequence of 7–9 bp, was designed to differentiate samples into groups. The amplified PCR products were determined by gel electrophoresis. PCR clean-up was performed with SPRIselect beads (Beckman Coulter Genomics). Samples with different inner tags were equimolarly pooled based on fluorometrically measured concentration using Qubit® dsDNA HS Assay Kit (Invitrogen™, USA) and microplate reader (Synergy Mx, BioTek, USA). Pools were used as a template for a second PCR with Nextera XT indexes (Illumina, USA). Differently indexed samples were checked and quantified using the three methods: qPCR using LightCycler 480 Instrument (Roche, USA) and KAPA Library Quantification Complete Kit (Roche, USA); 2100 Bioanalyzer Instrument using the High Sensitivity D1000 ScreenTape (Agilent Technologies, USA) and microplate reader (Synergy Mx, BioTek, USA) Qubit® using dsDNA HS Assay Kit (Invitrogen™, USA).  Samples were equimolarly pooled according to the measured concentration. The prepared library was checked with the same methods and concentration was measured shortly prior sequencing. The final library was diluted to a concentration of 8 pM and 20 % of PhiX DNA (Illumina, USA) was added. Sequencing was performed with the Miseq reagent kit V2 using a MiSeq instrument according to the manufacturer's instructions (Illumina, USA).

*Data processing*

Paired reads from 16s rRNA sequencing were first processed using an in-house pipeline implemented in Python 3. Steps of processing included trimming of low-quality 3' ends of reads, removal of read pairs containing unspecified base N and removal of pairs containing very short reads. In order to minimize sequencing and PCR-derived error, forward and reverse reads were denoised using the DADA2 amplicon denoising R package (2). Following denoising, the forward and reverse reads were joined into a single longer read using the fastq-join read joining utility (3). In order to be joined, reads in pairs had to have an overlap of at least 20 base pairs with no mismatches allowed. Pairs in which this was not the case were discarded. As the final step, chimeric sequences were removed from the joined reads using the remove Bimera function of the DADA2 R package. Subsequent taxonomic assignment was conducted by the uclust-consensus method from the QIIME (4) microbial analysis framework using the Silva v. 123 (5) reference database. In all the 90 samples, we identified 62,683

ASVs (amplicon sequence variant) and we detected 10 phyla, 19 classes, 24 orders, 44 families and 144 genera. The median sequencing coverage was 22,957 ASV per sample (min 7,385; max 38,528).

**Determination of short-chain fatty acids in serum**

SCFAs were analyzed in plasma by LC-MS according to a method described before [32] (6) with minor modifications. Briefly, fifty microliters of a mixed standard solution containing 4 mM of formic acid and acetic acid, 2 mM of propionic acid, and 1 mM of each of the other six SCFAs were added to a 2 mL borosilicate test tube that contained 1 mg of $^{13}$C6-3NPH HCl. Twenty-five microliters of 120 mM EDC-6% pyridine solution and twenty-five microliters 75% MeOH were then added to the mixture. The mixture was reacted at 4°C for 4 hours. Twenty-five microliters quinic acid in MeOH was added and quenching proceeded for 45 min. After quenching, the mixture was transferred to a volumetric flask with 10% MeOH and diluted with the same solvent to 100 mL. This solution was used as the internal standard mix and was stored in aliquots at -20°C. In total, 10 µl plasma was incubated with 60 µl 75% methanol, 10 µl 200 mM 3-NPH and 10 µl 120 mM EDC-6% pyridine at ambient temperature for 45 min with shaking. The reaction was quenched by addition of 10 µl of 200 mM quinic acid (15 min with shaking). The samples were centrifuged at 15 000 g for 5 min and the supernatant moved to a new tube. The samples were made up to 1 mL by 10% methanol in water and again centrifuged at 15 000 g for 5 min. In total, 100 µl of the derivativesed ($^{12}$C 3NPH) sample was mixed with 100 µl of labelled ($^{13}$C 3NPH) internal standard. A mixed external standard solution containing 3,2 µM – 0,63 nM of formic acid and acetic acid, 3,2 µM – 0,31 nM of propionic acid, and 0,8 µM – 0,16 nM of each of the other six SCFAs were always prepared fresh and used for each batch. Samples were analyzed by a 6500+ QTRAP triple-quadrupole mass spectrometer (AB Sciex, 11432 Stockholm, Sweden) which was equipped with an APCI source and operated in the negative-ion mode. Chromatographic separations were performed on a Phenomenex Kinetix Core-Shell C18 (2.1, 100 mm, 1.7 um 100Å) UPLC column with SecurityGuard ULTRA Cartridges (C18 2.1mm ID) (changed at regular intervals at). The column was backflushed for 60 min between each batch to ensure good chromatographic separation. Water (100% solvent A) and acetonitrile (100% solvent B) was the mobile phases for gradient elution. The column flow rate was 0.4 mL/min and the column temperature was 40°C, the autosampler was kept at 4°C. LC starting conditions at 0.5% B, held for 3 min, 3 min 2.5% B ramping linearly to 17% B at 6 min, then to 45% B at 10 min and 55% B at 13 min. Followed by a flush (100% B) and recondition (0.5% B), total runtime 15 min. The MRM transitions were optimized for the analytes one by one by direct infusion of the derivatives containing 10 µM of each fatty acid, essentially as according to Han et al. (7). The Q1/Q3 pairs were used in the MRM scan mode to optimize the collision energies for each analyte, and the two most sensitive pairs per analyte were used for the subsequent analyses. The retention time window for the scheduled MRM was 1 min for each analyte. The two MRM transitions per analyte, the Q1/Q3 pair that showed the higher sensitivity was selected as the MRM transition for quantitation. The other transition acted as a qualifier for the purpose of verification of the identity of the molecule. UPLC/MRM-MS data was acquired in the "scheduled MRM" mode using the Analyst 1.5 software and data processing was performed using the MultiQuant 3.0.3 software (AB Sciex, 11432 Stockholm, Sweden).

**Volatile compounds analysis of feces**

Eighty-seven samples (56 vegans, 31 omnivores) were analyzed. Stool was homogenized and diluted to equivalent of 1% (*w/w*) dry mass. This was pippeted into a 10 mL vial for headspace analysis, and prior sealing with a magnetic cap, 20 µl of sodium azide water solution (0.2%, *w/v*) was added as a bacteriostatic agent. Volatiles fingerprinting was performed using an Agilent 7890B gas chromatograph coupled to Leco Pegasus 4D time of flight mass

spectrometer. The instrument was equipped with a multi-purpose autosampler (MPS, Gerstel, USA), performing heated incubation, steering, and volatiles collection onto a solid-phase microextraction fiber with a divinylbenzen/carboxen/ polydimethylsiloxan (DVB/CAR/PDMS 50/30 µm) coating from Supelco (USA).

The sample was incubated and volatiles extracted onto a fiber stationary phase at 60 °C for 10 min and 20 minutes, respectively. Separation was performed on GC capillary column HP-Innowax (30 m × 0.25 mm i.d., 0.25 µm film thickness; Agilent Technologies, USA) with splitless injection at 250 °C. The GC oven temperature program was as follows: 40 °C for 1 min; then ramped at a rate of 10 °C/min to 180 °C; then at 20 °C/min to 260 °C and held for 2 min for a total GC run time of 21 min.

Time of flight mass spectrometer was operated with acquisition speed of 10 Hz to obtain full spectral information in a mass range 35–350 Da. Initial data processing (peak find and mass spectral deconvolution algorithms) and peak alignment was carried out using ChromaTOF software (LECO, USA). Compounds with a quantification mass (automatically selected unique mass for deconvoluted signal) signal to noise ratio (S/N), higher than 50 and present in more than half number of smallest sample class, were selected for alignment. For compounds to be listed in the aligned table, spectral similarity of two spectra at the same retention time (retention time difference in maximum 5 s) in different samples had to be more than 60 %. In the aligned table, areas of quantification masses for each aligned compound with tentative identification (based on spectral similarity of deconvoluted spectrum and NIST library) were provided. Further confirmation of signals identity was performed using comparison of measured retention index and retention indexes listed in the NIST library. An aligned table was exported to Microsoft Excel, where constant sum normalization was performed. Thus each compounds quantification mass area was divided by sum of all signals quantification mass areas in respective sample. Relative abundancies of compounds were used for statistical analysis.

**NMR analysis**

Ninety-five samples of serum (62 vegan, 33 omnivore), ninety-two samples of urine (62 vegan, 30 omnivore) and eighty-six samples of stool (56 vegan, 30 omnivore) were analysed. Fecal extracts were prepared from homogenised stool aliquot corresponding to 1.5% of dry mass. The homogenate was diluted with 400 µl of 5xPBS (200 µl if the dry mass of homogenate was less than 2.5%). The samples were sonicated for 1 minute at max. amplitude and cycles (UP200S, Heischler Ultrasound Technology), and the redistilled water up to the 2 ml was added (up to the 1 ml if the dry mass of homogenate was less than 2.5%). Samples were vortexed and centrifuged for 20 min at 20,000 g and 4°C. Supernatant was filtered through 0.2 µm filter and stored at -50°C until analysed.

Fecal extracts and urine samples were thawed at room temperature and then centrifuged at 14,000 rpm for 5 minutes at 5 °C. Aliquot of 540 µl urine was mixed with 60 µl phosphate buffer (1.5M $KH_2PO_4$ in $D_2O$ containing 2 mM $NaN_3$ and 0.1 % trimethylsilyl propionic acid (TSP), pH 7.4) and transferred into 5mm NMR tube. Aliquot of 360 µl fecal extract was mixed with 40 µl 0.1 % TSP in $D_2O$; 180 µl mixture was transferred into 3mm NMR tube. Aliquot of 220 µl serum sample was mixed with 440 µl cold methanol. The mixture was kept in freezer at -20°C for 30 minutes and then centrifuged at 14,000 rpm for 10 minutes at 4°C. The supernatant was transferred into fresh vial and vacuum dried. Evaporated supernatant was dissolved in 450 µl $D_2O$ with 50 µl 1.5 M phosphate buffer and 50 µl 0.1 % TSP, and then transferred into 5mm NMR tube.

NMR data were acquired on a 600 MHz Bruker Avance III spectrometer (Bruker BioSpin, Rheinstetten, Germany) equipped with a 5mm TCI cryogenic probe head. All experiments were performed using Topspin 3.5 software at 300 K with automatic tuning and matching, shimming and adjusting 90° pulse length for each sample. Proton spectra of urine and fecal samples were acquired using 1D-NOESY pulse sequence noesygppr1d with following acquisition parameters: number of scans NS=64 (urine) or 256 (fecal extracts), spectral width SW=20 ppm, 64k of data points (TD), relaxation delay for water presaturation d1=4 s. Serum data were analyzed from Carr-Purcell-Meiboom-Gill (CPMG) spectra acquired by cpmgpr1d pulse sequence with NS=192, SW=20 ppm, TD=64k, d1=4 s, echo time 0.3 ms, loop for T2 filter 126. *J*-resolved experiment (NS=2 for urine and serum or 8 for fecal extracts, SW=16,

TD=8k, number of increments=40, SW=78.125 Hz in the indirect dimension, d1=2 s for urine and serum or 4 s for fecal extracts) was performed on each sample to facilitate metabolite identification and quantitation in the case of signal overlap. Additional heteronuclear single quantum correlation (HSQC) and total correlation spectroscopy (TOCSY) experiments were executed for selected samples.

Acquired data were processed with Topspin 3.5 software. 1D-NOESY and CPMG spectra were line broadened (0.3 Hz), and together with 1D projections of *J*-resolved spectra were automatically phased, baseline corrected and referenced to the signal of TSP. The regions with signal of water, urea (in urine) and methanol (in serum extracts) were excluded and then spectra were normalized using probabilistic quotient normalization (PQN) method (8) to the pooled group of omnivores. Individual metabolites were identified using Chenomx software (Chenomx Inc., Edmonton, AB, Canada) and their proton and carbon data were then compared with the HMDB database (9). Metabolite concentrations were expressed as normalized intensities of corresponding signals in 1D-NOESY (for urine), CPMG (for serum extracts) and 1D projections of *J*-resolved (for fecal extracts) spectra.

**Bile acid analysis in feces**

One milliliter of methanol (LC/MS grade, Honeywell) was added to 50 mg of the lyophilized feces samples to extract BAs. The samples were sonicated (amplitude 50%, 15 s on, 10 s off, 4 cycles, on ice; Sonoplus HD3100, Bandelin) and then heated to 60°C for 45 minutes in the heat block (Thermo-shaker TS-100, Biosan). After cooling to room temperature, the samples were centrifuged at 1600 g for 10 min at 15°C and the supernatants were collected. To the precipitates, the same volume of methanol as in the first extraction step was added and mixed vigorously by vortex for 1 min. The samples were centrifuged at 20000 g for 10 min at 2°C and supernatants were collected. This extraction step was repeated once more. To the pooled extracts, a mix of labeled BAs (10 nM) was added as internal standard. Samples were centrifuged at 20000 g for 10 min at 2°C. For biochemical analysis, 50 µl of pooled extracts in methanol (described above) were used and diluted 1:1 with $dH_20$.

Liquid chromatography (LC) separation was performed using 1290 Infinity LC (Agilent Technologies) with a gradient elution from a Pinnacle DB C18 column (1.9 µm, 100 mm x 2.1 mm; Restek) and maintained at 55°C and a flow rate of 300 µl/min. The autosampler was kept at 10°C. The sample injection volume was 2 µl. Solvent A was water (LC/MS grade, VWR International) containing 10 mM $NH_4F$. Solvent B was 100 % methanol (LC/MS grade, Honeywell). The gradient was changed linearly and started with 40% solvent B at 0 min with increasing to 57% within 3.5 min, then 59% until 9.5 min, then 70% until 10 min, then 72% until14min, 76% until 16 min and at last 95% for 9 minutes with 5 min post run for column equilibration with 40% methanol. The column eluent was introduced into the MS.

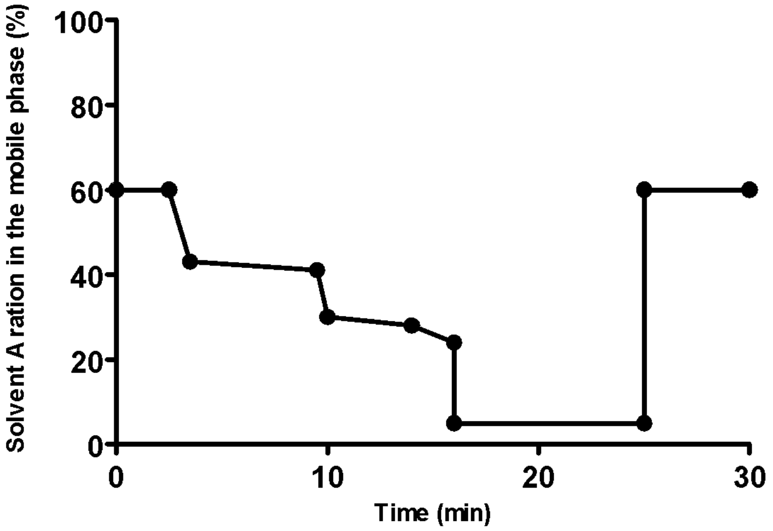


MS analysis was performed using 6550 iFunnel Q-TOF LC/MS (Agilent Technologies) equipped with a Dual AJS ESI probe in negative-ion mode. A capillary voltage of -3500 V, a gas temperature of 120°C, and a sheath gas temperature of 350°C were used. The nozzle voltage was 500 V, the drying gas and sheath gas flow were 14 l/min and 11 l/min, respectively.

The m/z values and retention time values of BAs were investigated in a preliminary experiment within the same experimental setup by three successive analyses of single standard mixture samples.

System control and data acquisition were performed by Agilent MassHunter Quadrupole Time of Flight Acquisition Software (B.06) with Qualitative Analysis (B.07 SP2) Software. Data were analyzed using Find by Formula and database search software functions. Acceptance criteria included a match to retention time (within 0.02 minutes), isotope spacing and abundance, accurate mass (within 5 ppm), MS spectral database matching, and overall score (>75). Concentrations of individual BAs were calculated from the peak areas detected in chromatogram relative to the internal standard, each BA with its own deuterium or C13 labeled BA (Cambridge Isotopes).

**Data analysis**

*Identification of discriminating features between cohorts*

Aiming to identify the most relevant discriminating features, we applied both univariate and multivariable statistical analyses. Univariate statistical analyses were performed by Mann-Whitney-Wilcoxon test. Results were considered significant at $P<0.05$ and at false discovery rate <0.1. Multivariable analyses, partial least squares-discriminant analysis (PLS-DA) was performed to unravel which combination of parameters would allow for the most accurate discrimination of vegans and omnivores. For each dataset (e.g., microbiome, clinical data, etc.), we created new PLS-DA classifier using all features in

particular dataset. The goodness of fit was estimated according to the $R^2Y$ value, the predictive power ($Q^2$) was obtained using 10-fold cross-validation approach.

*Classification into patients groups using machine learning*
We analyzed the discriminating power of each omics dataset using machine learning; specifically, we used a random forest method. First, we performed a feature selection on each dataset by selecting only features with false discovery rate < 0.1. Then we run the random forest method with 10-fold cross-validation on the filtered data. We used the R package *caret* [10] for training the random forest method, which also performs auto-tuning of the parameters (mtry - number of random features sampled at each split node). The number of trees was kept constant at 500.

The validity of a trained model was verified using permutation test with 300 repetitions. That is, in each repetition, the assignment of patients to groups is randomized, and a model is trained on such randomized dataset. An empirical p-value is calculated as the percentage of repetitions for which the accuracy is larger than the accuracy obtained using the original non-randomized data.

*Network analysis*
We constructed correlation networks based on Spearman's correlation coefficient to analyse the correlations between the datasets. To create a correlation network between datasets A and B, the following steps were done

1. Compute the pairwise Spearman's correlation coefficient (ρ) between each pair of variables from A and B.
2. Select statistically significant correlations ($p<0.5$)
3. Select correlations with $|\rho| > \rho_{AB}$
4. Select statistically significant pairwise correlations between variables of A having $|\rho| > \rho_{AA}$ such that at least one of the variables was selected in step 3.
5. Select statistically significant pairwise correlations between variables of B having $|\rho| > \rho_{BB}$ such that at least one of the variables was selected in step 3.

The following table shows the thresholds for the correlation coefficients

| A | B | $\rho_{AB}$ | $\rho_{AA}$ $\rho_{BB}$ | $\rho_{BB}$ |
|---|---|---|---|---|
| SCFA | microbiome | 0.25 | 0.3 | 0.6 |
| SCFA | NMR serum | 0.3 | 0.3 | 0.5 |
| SCDA | diet | 0.2 | 0.3 | 0.3 |
| Serum metabolome | diet | 0.2 | 0.6 | 0.3 |
| Serum metabolome | microbiome | 0.35 | 0.6 | 0.7 |
| VOC | microbiome | 0.5 | 0.7 | 0.7 |

| | | | | |
|---|---|---|---|---|
| fecal metabolome | microbiome | 0.5 | 0.7 | 0.8 |
| Fecal metabolome | diet | 0.3 | 0.7 | 0.3 |
| microbiome | diet | 0.25 | 0.7 | 0.3 |
| VOC | diet | 0.3 | 0.7 | 0.3 |
| Fecal metabolome | Serum metabolome | 0.34 | 0.7 | 0.7 |

**Supplemental references**


1. Klindworth, A. *et al*. Evaluation of general 16S ribosomal RNA gene PCR primers for classical and next-generation sequencing-based diversity studies. Nucleic Acids Res. 41, e1 (2013).
2. B. J. Callahan, P. J. McMurdie, M. J. Rosen, A. W. Han, A. J. A. Johnson, S. P. Holmes, DADA2: High-resolution sample inference from Illumina amplicon data, Nat Methods 13, 581–583 (2016).
3. Aronesty,E. Comparison of sequencing utility programs. Open Bioinform. J., 7, 1–8 (2013).
4. J. G. Caporaso, J. Kuczynski, J. Stombaugh, K. Bittinger, F. D. Bushman, E. K. Costello, N. Fierer, A. G. Peña, J. K. Goodrich, J. I. Gordon, G. A. Huttley, S. T. Kelley, D. Knights, J. E. Koenig, R. E. Ley, C. A. Lozupone, D. McDonald, B. D. Muegge, M. Pirrung, J. Reeder, J. R. Sevinsky, P. J. Turnbaugh, W. A. Walters, J. Widmann, T. Yatsunenko, J. Zaneveld, R. Knight, QIIME allows analysis of high-throughput community sequencing data, Nat Methods 7, 335–336 (2010).
5. Quast, C. et al. The SILVA ribosomal RNA gene database project: improved data processing and web-based tools. Nucleic Acids Res. 41, D590–D596 (2013).
6. citace 32 Landberg
7. Han, J., Lin, K., Sequeira, C. & Borchers, C.H. An isotope-labeled chemical derivatization method for the quantitation of short-chain fatty acids in human feces by liquid chromatography–tandem mass spectrometry. *Analytica chimica acta* **854**, 86-94 (2015).
8. F. Dieterle, A. Ross, G. Schlotterbeck, H. Senn, Probabilistic quotient normalization as robust method to account for dilution of complex biological mixtures. Application in 1H NMR metabonomics. Anal Chem. 78(13), 4281-4290 (2006)
9. D. S. Wishart *et al*. HMDB 4.0: the human metabolome database for 2018. Nucleic Acids Res. 46(D1), D608-D617 (2018)
10. Kuhn, M. Caret package. Journal of Statistical Software 28(5), (2008)

# 2 Supplementary Figures and Tables

**Table S1** *EMP primer, overhang and tag sequences*

| | | | |
|---|---|---|---|
| 16S_EMP-F primer | GTGYCAGCMGCCGCGGTAA | 16S_EMP-R primer | GGACTACNVGGGTWTCTAAT |
| 16S_EMP-F overhang | TCGTCGGCAGCGTCAGATGTGTATAAGAGACAG | 16S_EMP-R overhang | GTCTCGTGGGCTCGGAGATGTGTATAAGAGACAG |
| 16S_EMP-F_tag1 | AGCCTTCGTCGC | 16S_EMP-R_tag1 | CCTAACGGTCCA |
| 16S_EMP-F_tag2 | TCCATACCGGAA | 16S_EMP-R_tag2 | CGCGCCTTAAAC |
| 16S_EMP-F_tag3 | AGCCCTGCTACA | 16S_EMP-R_tag3 | TATGGTACCCAG |
| 16S_EMP-F_tag4 | TGAGACCCTACA | 16S_EMP-R_tag4 | GCCTCTACGTCG |
| 16S_EMP-F_tag5 | ACTTGGTGTAAG | 16S_EMP-R_tag5 | ACTACTGAGGAT |
| 16S_EMP-F_tag6 | ATTACGTATCAT | 16S_EMP-R_tag6 | AATTCACCTCCT |
| 16S_EMP-F_tag7 | CACGCAGTCTAC | 16S_EMP-R_tag7 | CGTATAAATGCG |
| 16S_EMP-F_tag8 | TGTGCACGCCAT | 16S_EMP-R_tag8 | ATGCTGCAACAC |
| 16S_EMP-F_tag9 | CCGGACAAGAAG | 16S_EMP-R_tag9 | ACTCGCTCGCTG |
| 16S_EMP-F_tag10 | TTGCTGGACGCT | 16S_EMP-R_tag10 | TTCCTTAGTAGT |
| 16S_EMP-F_tag11 | TACTAACGCGGT | 16S_EMP-R_tag11 | CGTCCGTATGAA |
| 16S_EMP-F_tag12 | GCGATCACACCT | 16S_EMP-R_tag12 | ACGTGAGGAACG |
| 16S_EMP-F_tag13 | CAAACGCACTAA | 16S_EMP-R_tag13 | GGTTGCCCTGTA |
| 16S_EMP-F_tag14 | GAAGAGGGTTGA | 16S_EMP-R_tag14 | CATATAGCCCGA |
| 16S_EMP-F_tag15 | TGAGTGGTCTGT | 16S_EMP-R_tag15 | GCCTATGAGATC |
| 16S_EMP-F_tag16 | TTACACAAAGGC | 16S_EMP-R_tag16 | CAAGTGAAGGGA |

**Table S2** *Sequences of degenerate primers and their target bacteria.*

| Cluster | Bacterial genome | Primer Sequence (5´to 3´) |
|---|---|---|
| A | *Flavonifractor plautii* strain 2789ST<br>*Flavonifractor plautii* strain 2789S<br>*Pseudoflavonifractor capillosus* ATCC | Forward: MCTGGGYATYCACACCGAG; Reverse: GGTGGGCGATGGAGATAA |
| B | *Clostridium propionicum* DSM 1682 g<br>Coprococcus eutactus strain 2789S<br>Coprococcus eutactus strain 2789STD<br>Coprococcus eutactus strain 2789STDY5<br>Coprococcus eutactus strain 2789STD<br>Lachnospiraceae bacterium MD335<br>Lachnospiraceae bacterium A4 acPFC | Forward: GGKCCBATHGARRTTGCAGA; Reverse: TKTCGTCMASCCABTCATAC |
| C | Faecalibacterium prausnitzii A2-165<br>Faecalibacterium prausnitzii strain A21<br>Faecalibacterium prausnitzii strain Ind<br>Faecalibacterium prausnitzii strain 942<br>Faecalibacterium cf. prausnitzii K<br>Faecalibacterium prausnitzii<br>Clostridium sp. M62/1<br>Clostridium symbiosum WAL-14673<br>Clostridium symbiosum ATCC 14940<br>Eubacterium maltosivorans strain YI c<br>Eubacterium limosum strain SA11 comp<br>Eubacterium limosum strain ATCC 8486<br>Eubacterium callanderi strain FD g | Forward: GBGACTGGSTRGATTAYG; Reverse: TCVACRTACATYTCSGTGTG |
| D | Lachnospiraceae bacterium 3-1 acPFp<br>Lachnospiraceae bacterium A2 acPFL-s | Forward: TGGAAYTCMTGGCATATGTC ; Reverse: VGMRTTGTTRATGGAMATAAA |

| | | |
|---|---|---|
| | | |
| | Lachnospiraceae bacterium 3-1 acPFp-s | |
| | Roseburia intestinalis L1-82 | |
| | Roseburia inulivorans DSM 16841 | |
| E | Lachnospiraceae bacterium TF01-11 | Forward: TGHAGSABHTSWTTTTACATGGA; Reverse: SSCTTTGCAATGTCAACAAA |
| | Clostridiales bacterium KA00134 HMPR | |
| | Anaerobutyricum hallii DSM 3353 | |
| F | Anaerostipes caccae DSM 14662 | Forward: AAATATGCCTCGHTGCYTWG; Reverse: ARRTARGCACCYAWAACGAAATC |
| | Anaerostipes hadrus DSM 3319 | |
| | Clostridium sp. SS2/1 | |
| | Clostridium sp. JN-9 | |

Bacterial genomes were divided into clusters according to their phylogenetic distance. In the same cluster, one primer pair covers all *but* gene variants.
K = G or T; B = C or G or T; H = A or C or T; R = A or G; M = A or C; S = G or C; Y = C or T; V = A or C or G; W = A or T (as stated by IUPAC nucleotide code).

**Table S3** *List of quantified metabolites in urine. serum, and fecal extracts using NMR (with corresponding $^{1}H$ and $^{13}C$ chemical shifts).*

| | **metabolite** | **$^{1}$H chemical shift [ppm]** | **$^{13}$C chemical shift [ppm]** | **source** |
|---|---|---|---|---|
| 1. | Lipoproteins $CH_3$- | **0.80 – 0.87** | 16.8 | S |
| 2. | Valerate | **0.86** (t). 1.30 (m). 1.53 (m). 2.18 (t) | 24.6. 30.8. 40.2 | F |
| 3. | 3-Methyl-2-oxovalerate | 0.89 (t). **1.10** (d) | 13.3. 16.4 | S |
| 4. | Butyrate | **0.90** (t). 1.56 (m). 2.16 (t) | 16.0. 22.2. 42.4 | F |
| 5. | 2-Hydroxybutyrate | **0.90** (t). 1.66 (m). 4.00 (m) | 11.4 | S |
| 6. | 2-Oxoisocaproate | **0.94** (d). 2.61 (d) | 24.5. 51.0 | S |
| 7. | Isoleucine | 0.94 (t). **1.01** (d). 1.27 (m). 1.47 (m). 3.68 (d) | 13.9. 17.4. 27.2. 38.6. 62.3 | S. F |
| 8. | Leucine | **0.96** (d). 0.97 (d). 1.71 (m). 1.72 (m). 3.73 (m) | 23.7. 24.8. 26.9. 42.6. 56.2 | S. F |
| 9. | Valine | 0.99 (d). **1.05** (d). 2.27 (m). 3.62 (d) | 19.4. 20.7. 31.8. 63.1 | U. S. F |
| 10. | Propionate | **1.06** (t). 2.19 (q) | 13.0. 33.5 | F |
| 11. | 3-Hydroxyisobutyrate | **1.07** (d). 2.49 (m). 3.54 (m). 3.70 (m) | 16.8 | U. S |
| 12. | 2-Oxoisovalerate | **1.12** (d) | 19.1 | S |
| 13. | Ethanol | 1.19 (t). **3.65** (q) | n.d. | S. F |
| 14. | 3-Hydroxybutyrate | **1.20** (d). 2.31 (dd). 2.40 (dd) | 24.5. 68.4 | S |
| 15. | 3-Hydroxyisovalerate | **1.28** (s) | 30.9 | U |
| 16. | Lactate | 1.33 (d). **4.11** (q) | 22.9. 71.2 | S |
| 17. | Threonine | 1.33 (d). 3.59 (d). **4.25** (m) | 22.3. 63.2. 68.9 | S |
| 18. | 2-Hydroxyisobutyrate | **1.36** (s) | 29.6 | U |
| 19. | Lysine | 1.45 (m). 1.52 (m). 1.73 (m). 1.91 (m). **3.04** (t) | 24.2. 29.1. 32.7. 41.8 | S. F |
| 20. | Alanine | **1.49** (d). 3.79 (q) | 18.9. 53.3 | U. S. F |
| 21. | Acetate | **1.92** (s) | 26.1 | S. F |
| 22. | Ornithine | 1.95 (m). **3.06** (t) | 30.3. 41.5 | S |
| 23. | Proline | 2.01 (dd). 2.08 (m). 2.36 (m). 3.43 (m). **4.14** (m) | 26.5. 31.8. 48.8. 63.9 | S |
| 24. | Glutamate | 2.04 (m). **2.34** (m). 2.37 (m) | 29.8. 36.8 | F |
| 25. | Glutamine | 2.14 (m). **2.46** (m) | 29.1. 33.6 | S |

| | | | | |
|---|---|---|---|---|
| 26. | Methionine | 2.14 (s). **2.65** (t). 3.85 (dd) | 16.7. 31.6. 56.8 | F |
| 27. | Acetone | **2.23** (s) | n.d. | S |
| 28. | p-Cresyl sulfate | **2.34** (s). 7.21 (m). 7.28 (m) | 22.8. 124.2. 133.0 | U |
| 29. | Pyruvate | **2.38** (s) | n.d. | S |
| 30. | Succinate | **2.41** (s) | 37.0 | F |
| 31. | Citrate | 2.55 (d). **2.69** (d) | 48.5 | U. S |
| 32. | Aspartate | 2.68 (dd). **2.82** (dd). 3.90 (m) | 39.4. 55.1 | F |
| 33. | Dimethylamine | **2.72** (s) | 37.4 | U. S |
| 34. | Asparagine | **2.87** (dd). 2.95 (dd). 4.00 (m) | 37.5. 54.0 | S |
| 35. | Trimethylamine | **2.91** (s) | 47.6 | F |
| 36. | Creatinine | **3.05** (s). 4.06 (s) | 33.0 | U |
| 37. | Dimethylsulfone[x] | **3.16** (s) | n.d. | S |
| 38. | Trimethylamine N-oxide | **3.26** (s) | 62.4 | U |
| 39. | Methanol | **3.37** (s) | 51.7 | F |
| 40. | Tyrosine | 3.06 (dd). 3.21 (dd). 3.95 (dd). **6.91** (m). 7.20 (m) | 38.3. 58.8. 118.6. 133.5 | S. F |
| 41. | Phenylalanine | 3.13 (dd). 3.29 (dd). 4.00 (m). 7.34 (m). 7.38 (m). **7.44** (m) | 39.1. 58.8. 130.4. 131.8. 132.2 | U. S. F |
| 42. | Glycine | **3.57** (s) | 44.2 | U. S. F |
| 43. | Hippurate | 3.98 (d). 7.55 (m). **7.64** (m). 7.84 (m) | 46.7. 130.0. 131.7. 135.1 | U |
| 44. | Histidine | 3.99 (dd). **7.10** (s). 7.90 (s) | 57.4. 119.8. 138.9 | S |
| 45. | Tryptophan | 4.06 (dd). 7.21 (m). 7.29 (m). 7.33 (m). 7.55 (m). **7.74** (m) | 29.2. 57.8. 114.7. 121.2. 122.2. 124.8. 127.9 | S |
| 46. | Trigonelline | 4.44 (s). 8.84 (m). **9.12** (s) | n.d. | U |
| 47. | 1-Methylnicotinamide | 4.48 (s). 8.90 (m). 8.96 (m). **9.27** (s) | n.d. | U |
| 48. | Arabinose | **4.52** (d). 5.25 (d) | 99.6 | F |
| 49. | Glucose | 4.66 (d). **5.25** (d)<br>3.26 (dd). 3.41 (m). 3.37 (m). 3.50 (dd). 3.55 (dd). 3.72 (m). 3.77 (dd). 3.84 (m). 3.90 (dd) | 94.8. 98.6<br>63.3. 63.5. 72.3. 74.2. 75.5. 76.9. 78.5. 78.7 | U. S. F |
| 50. | Mannose | 4.90 (d). **5.18** (d) | 96.4. 96.7 | S |
| 51. | Ribose | **4.94** (d). 5.26 (d). 5.39 (d) | 96.5. 99.0. 103.8 | F |

| | | | | |
|---|---|---|---|---|
| 52. | Uracil | 5.81 (d). **7.54** (d) | 103.9. 146.3 | F |
| 53. | Fumarate | **6.52** (s) | n.d. | F |
| 54. | 3-Indoxylsulfate | 7.28 (m). 7.36 (m). **7.50** (m). 7.70 (m) | 115.0. 118.8. 120.2. 125.3 | U |
| 55. | Formate | **8.46** | n.d. | S. F |
| 56. | Urocanate | **6.39** (d). 7.31 (d). 7.39 (br s). 7.84 (br s) | 124.1. 124.6. 133.8. 140.6 | F |
| 57. | Isovalerate | **0.91** (d). 2.06 (d) | n.d. | F |
| 58. | Glycerol | 3.56 (dd). **3.66** (dd) | 65.3 | S |
| 59. | Unidentified N-acetyl | **2.07** (s) | 24.9 | S |
| 60. | Unidentified | **4.42** (s) | n.d. | F |
| 61. | Unidentified | **3.11** (s) | 55.7 | F |
| 62. | Unidentified | **0.80** (br t) | n.d. | F |
| 63. | Unidentified | **1.43** (d) | n.d. | S |
| 64. | Unidentified | **1.11** (d). 4.11 | 18.4 | U |

The table lists all metabolites quantified in urine (U). serum (S). and fecal extracts (F); the signals used for metabolite quantification are in bold. Signal multiplicity is marked as follows: (s)-singlet. (d)-doublet. (t)-triplet. (dd)-doublet od doublet. (q)-quartet. (m)-multiplet; n.d. – signals not detected; $^{x}$-tentative assignment.

**Table S4** *Normalized diversity*.

| | observed species | Chao1 | Pielou | Shannon | Simpson |
|---|---|---|---|---|---|
| VG | 360 (195)* | 239 (111) | 0.7 (0.1) | 5.1 (1.1) | 0.93 (0.08)* |
| O | 453 (206) | 294 (108) | 0.7 (0.1) | 5.7 (0.9) | 0.96 (0.05) |

The calculation of diversity was normalized per 7.000 reads per sample. Data are given as median (interquartile range). * $p < 0.05$ vs O

**Table S5** *Gut microbiota composition*

| | VG | | | O | | | p-value | FDR | Cliff´s | VIP | UDAA | PLS-DA | prevalence (%) | |
|---|---|---|---|---|---|---|---|---|---|---|---|---|---|---|
| | median | Q1 | Q3 | median | Q1 | Q3 | | | delta | | | | VG | O |
| Lachnospira | **0.79** | 0.30 | 1.65 | **0.40** | 0.14 | 0.67 | 0.002 | 0.036 | -0.409 | 1.73 | X | X | 98 | 97 |
| Lachnospiraceae NK4A136 group | **0.65** | 0.24 | 1.60 | **0.70** | 0.20 | 1.77 | 0.546 | 0.728 | -0.079 | 1.14 | | X | 92 | 97 |
| Ruminiclostridium | **0.43** | 0.21 | 0.88 | **0.29** | 0.14 | 0.39 | 0.003 | 0.037 | -0.391 | 1.57 | X | X | 95 | 97 |
| Ruminococcaceae UCG-014 | **0.79** | 0.09 | 3.32 | **0.29** | 0.00 | 1.49 | 0.003 | 0.037 | -0.388 | 1.52 | X | X | 78 | 60 |
| Ruminococcaceae UCG-003 | **0.28** | 0.10 | 0.43 | **0.15** | 0.03 | 0.28 | 0.001 | 0.035 | -0.429 | 1.81 | X | X | 92 | 77 |
| Lachnospiraceae UCG-001 | **0.14** | 0.02 | 0.27 | **0.08** | 0.00 | 0.23 | 0.052 | 0.177 | -0.253 | 1.16 | | X | 78 | 73 |
| Lachnospiraceae UCG-005 | **0.06** | 0.00 | 0.24 | **0.00** | 0.00 | 0.01 | 0.000 | 0.019 | -0.473 | 2.17 | X | X | 67 | 33 |
| Haemophilus | **0.04** | 0.01 | 0.19 | **0.02** | 0.00 | 0.08 | 0.058 | 0.181 | -0.247 | 1.11 | | X | 75 | 67 |
| Lachnospiraceae NC2004 group | **0.02** | 0.00 | 0.12 | **0.01** | 0.00 | 0.03 | 0.096 | 0.251 | -0.217 | 1.05 | | X | 5 | 0 |
| Lachnospiraceae UCG-010 | **0.00** | 0.00 | 0.05 | **0.00** | 0.00 | 0.01 | 0.002 | 0.036 | -0.407 | 1.70 | X | X | 50 | 30 |
| Oxalobacter | **0.00** | 0.00 | 0.05 | **0.00** | 0.00 | 0.00 | 0.001 | 0.026 | -0.450 | 1.70 | X | X | 40 | 17 |
| Chloroplast | **0.00** | 0.00 | 0.02 | **0.00** | 0.00 | 0.00 | 0.002 | 0.037 | -0.394 | 1.47 | X | X | 35 | 17 |
| Prevotella 2 | **0.00** | 0.00 | 0.00 | **0.00** | 0.00 | 0.00 | 0.005 | 0.047 | -0.367 | 1.02 | X | X | 10 | 3 |
| Erysipelotrichaceae UCG-004 | **0.00** | 0.00 | 0.00 | **0.00** | 0.00 | 0.00 | 0.006 | 0.048 | -0.360 | 1.59 | X | X | 8 | 0 |
| Acetitomaculum | **0.00** | 0.00 | 0.00 | **0.00** | 0.00 | 0.00 | 0.010 | 0.075 | -0.333 | 1.31 | X | X | 20 | 10 |
| Tyzzerella 3 | **0.00** | 0.00 | 0.13 | **0.00** | 0.00 | 0.00 | 0.011 | 0.075 | -0.331 | | X | | 45 | 27 |
| Mitsuokella | **0.00** | 0.00 | 0.00 | **0.00** | 0.00 | 0.00 | 0.011 | 0.075 | -0.331 | 1.32 | X | X | 13 | 10 |
| Tyzzerella 3 | **0.00** | 0.00 | 0.13 | **0.00** | 0.00 | 0.00 | 0.011 | 0.075 | -0.331 | 1.63 | | X | 45 | 27 |
| Alloprevotella | **0.00** | 0.00 | 0.00 | **0.00** | 0.00 | 0.00 | 0.013 | 0.076 | -0.324 | 1.40 | X | X | 8 | 0 |
| Enterorhabdus | **0.00** | 0.00 | 0.05 | **0.00** | 0.00 | 0.00 | 0.016 | 0.089 | -0.312 | 1.24 | X | X | 35 | 23 |
| Prevotella 7 | **0.00** | 0.00 | 0.00 | **0.00** | 0.00 | 0.00 | 0.018 | 0.089 | -0.307 | | X | | 23 | 10 |
| Bacteroidales | **0.00** | 0.00 | 0.00 | **0.00** | 0.00 | 0.00 | 0.018 | 0.089 | -0.307 | | X | | 15 | 7 |
| Lachnospiraceae UCG-003 | **0.00** | 0.00 | 0.00 | **0.00** | 0.00 | 0.00 | 0.019 | 0.089 | -0.306 | 1.09 | X | X | 22 | 13 |
| Succinivibrio | **0.00** | 0.00 | 0.00 | **0.00** | 0.00 | 0.00 | 0.019 | 0.089 | -0.306 | 1.28 | X | X | 5 | 0 |
| Peptococcus | **0.00** | 0.00 | 0.00 | **0.00** | 0.00 | 0.00 | 0.023 | 0.101 | -0.297 | 1.23 | X | X | 25 | 13 |

| | | | | | | | | | | | | | | |
|---|---|---|---|---|---|---|---|---|---|---|---|---|---|---|
| Ruminococcaceae UCG-004 | **0.00** | 0.00 | 0.00 | **0.00** | 0.00 | 0.00 | 0.024 | 0.102 | -0.293 | 1.02 | X | X | 30 | 17 |
| Weissella | **0.00** | 0.00 | 0.00 | **0.00** | 0.00 | 0.00 | 0.033 | 0.124 | -0.278 | 1.45 | | X | 7 | 3 |
| Shuttleworthia | **0.00** | 0.00 | 0.00 | **0.00** | 0.00 | 0.00 | 0.057 | 0.181 | -0.248 | 1.08 | | X | 10 | 7 |
| NB1-n | **0.00** | 0.00 | 0.00 | **0.00** | 0.00 | 0.00 | 0.066 | 0.199 | -0.239 | 1.04 | | X | 12 | 7 |
| Lactobacillus | **0.00** | 0.00 | 0.00 | **0.00** | 0.00 | 0.00 | 0.072 | 0.210 | -0.234 | 1.02 | | X | 27 | 20 |
| Alistipes | **1.70** | 0.48 | 3.56 | **3.21** | 1.33 | 4.72 | 0.083 | 0.226 | 0.226 | 1.36 | | X | 90 | 100 |
| Bifidobacterium | **1.05** | 0.17 | 2.71 | **1.92** | 0.55 | 4.61 | 0.250 | 0.446 | 0.150 | 1.06 | | X | 93 | 100 |
| Blautia | **0.74** | 0.42 | 1.24 | **1.82** | 1.14 | 2.24 | 0.029 | 0.118 | 0.283 | 1.32 | | X | 100 | 100 |
| Fusicatenibacter | **0.26** | 0.15 | 0.62 | **0.64** | 0.33 | 1.05 | 0.098 | 0.251 | 0.216 | 1.14 | | X | 95 | 100 |
| Dorea | **0.27** | 0.10 | 0.49 | **0.58** | 0.36 | 0.70 | 0.023 | 0.101 | 0.296 | 1.39 | X | X | 92 | 100 |
| Anaerostipes | **0.21** | 0.11 | 0.52 | **0.42** | 0.19 | 1.00 | 0.362 | 0.573 | 0.119 | 1.13 | | X | 97 | 97 |
| Ruminococcaceae_uncultured | **0.24** | 0.11 | 0.38 | **0.40** | 0.31 | 0.77 | 0.042 | 0.148 | 0.264 | 1.39 | | X | 97 | 100 |
| [Eubacterium] hallii group | **0.15** | 0.04 | 0.34 | **0.39** | 0.18 | 0.63 | 0.012 | 0.076 | 0.326 | 1.54 | X | X | 92 | 97 |
| Peptoclostridium | **0.09** | 0.03 | 0.37 | **0.33** | 0.17 | 0.44 | 0.031 | 0.122 | 0.280 | 1.39 | | X | 83 | 97 |
| Odoribacter | **0.08** | 0.02 | 0.33 | **0.32** | 0.13 | 0.44 | 0.005 | 0.048 | 0.362 | 1.81 | X | X | 78 | 100 |
| Ruminiclostridium 5 | **0.08** | 0.03 | 0.14 | **0.23** | 0.13 | 0.39 | 0.004 | 0.044 | 0.374 | 1.83 | X | X | 87 | 100 |
| Erysipelotrichaceae UCG-003 | **0.09** | 0.04 | 0.25 | **0.22** | 0.16 | 0.56 | 0.019 | 0.089 | 0.306 | 1.41 | X | X | 93 | 100 |
| Streptococcus | **0.06** | 0.03 | 0.17 | **0.22** | 0.16 | 0.60 | 0.000 | 0.012 | 0.511 | 2.06 | X | X | 77 | 97 |
| Anaerotruncus | **0.03** | 0.00 | 0.15 | **0.16** | 0.05 | 0.49 | 0.001 | 0.036 | 0.420 | 2.06 | X | X | 70 | 93 |
| Ruminiclostridium 9 | **0.07** | 0.02 | 0.12 | **0.14** | 0.07 | 0.20 | 0.027 | 0.111 | 0.288 | 1.10 | | X | 88 | 97 |
| Escherichia-Shigella | **0.00** | 0.00 | 0.09 | **0.13** | 0.03 | 0.60 | 0.002 | 0.037 | 0.398 | 1.84 | X | X | 50 | 80 |
| [Ruminococcus] gauvreauii group | **0.03** | 0.00 | 0.07 | **0.11** | 0.01 | 0.21 | 0.019 | 0.089 | 0.304 | 1.21 | X | X | 60 | 77 |
| [Eubacterium] ventriosum group | **0.05** | 0.00 | 0.11 | **0.09** | 0.05 | 0.20 | 0.080 | 0.226 | 0.228 | 1.11 | | X | 75 | 90 |
| Ruminiclostridium 6 | **0.00** | 0.00 | 0.09 | **0.06** | 0.00 | 0.29 | 0.096 | 0.251 | 0.217 | 1.15 | | X | 45 | 67 |
| Family XIII UCG-001 | **0.01** | 0.00 | 0.03 | **0.05** | 0.02 | 0.10 | 0.004 | 0.044 | 0.377 | 1.65 | X | X | 60 | 87 |
| Coriobacteriaceae | **0.00** | 0.00 | 0.04 | **0.05** | 0.00 | 0.20 | 0.011 | 0.075 | 0.329 | 1.69 | X | X | 40 | 70 |
| Family XIII AD3011 group | **0.01** | 0.00 | 0.03 | **0.02** | 0.02 | 0.07 | 0.009 | 0.070 | 0.341 | 1.57 | X | X | 58 | 83 |
| Actinomyces | **0.00** | 0.00 | 0.01 | **0.01** | 0.00 | 0.04 | 0.080 | 0.226 | 0.228 | 1.10 | | X | 35 | 60 |
| Peptococcaceae | **0.00** | 0.00 | 0.00 | **0.00** | 0.00 | 0.03 | 0.005 | 0.047 | 0.366 | 1.94 | X | X | 12 | 50 |

| | | | | | | | | | | | | | |
|---|---|---|---|---|---|---|---|---|---|---|---|---|---|
| Pseudoflavonifractor | **0.00** | 0.00 | 0.00 | **0.00** | 0.00 | 0.01 | 0.774 | 0.851 | 0.038 | 1.01 | | X | 5 | 27 |
| Gordonibacter | **0.00** | 0.00 | 0.00 | **0.00** | 0.00 | 0.00 | 0.641 | 0.769 | 0.061 | 1.25 | | X | 3 | 27 |
| Victivallis | **0.00** | 0.00 | 0.00 | **0.00** | 0.00 | 0.01 | 0.569 | 0.745 | 0.074 | 1.18 | | X | 12 | 30 |
| Ruminococcaceae UCG-009 | **0.00** | 0.00 | 0.00 | **0.00** | 0.00 | 0.03 | 0.213 | 0.409 | 0.162 | 1.27 | | X | 18 | 43 |

Data are presented as compositional (proportion of the particular bacteria of total sum of bacteria) and are shown as medians (1st. 3rd quartile). Only genera selected by at least one statistical methods as significantly contributing to the groups' separation are shown. Green lines indicates metabolites enriched in vegans, grey lines indicates metabolites depleted in vegans compared with omnivores. UDAA, univariable differential abundance analysis; FDR, false discovery rate; PLS-DA, partial least squares discriminant analysis; VIP, variable importance in projection. VIP score of a variable measures its discriminatory power and is computed as root of sum of weighted explained sum of squares over all dimensions, divided by the cumulative explained sum of squares.

**Table S6** *Fecal metabolome composition*

| name | | detection | VG | | | O | | | | | | | | |
|---|---|---|---|---|---|---|---|---|---|---|---|---|---|---|
| | | method | median | Q1 | Q3 | median | Q1 | Q3 | pvalue | adjpvalue | cliff | VIP | UDAA | PLS-DA |
| Methanol | alcohol | NMR | **0,70** | 0,47 | 0,98 | **0,54** | 0,39 | 0,80 | 0,008 | 0,064 | -0,349 | 1,31 | X | X |
| Glucose | sacharides | NMR | **0,48** | 0,21 | 0,91 | **0,33** | 0,12 | 0,63 | 0,047 | 0,175 | -0,262 | 1,20 | | X |
| Arabinose | | NMR | **0,06** | 0,04 | 0,08 | **0,04** | 0,03 | 0,06 | 0,001 | 0,019 | -0,426 | 1,26 | X | X |
| Acetate | SCFA and SCFA derivatives | NMR | **64,11** | 58,19 | 68,02 | **64,05** | 57,70 | 66,68 | 0,052 | 0,175 | -0,256 | 1,39 | | X |
| Methyl acetate | | GC-MS | **0,02** | 0,00 | 0,13 | **0,00** | 0,00 | 0,02 | 0,494 | 0,697 | -0,090 | 1,47 | | X |
| Butyl acetate | | GC-MS | **0,27** | 0,00 | 0,78 | **0,00** | 0,00 | 0,28 | 0,500 | 0,700 | -0,089 | 1,28 | | X |
| Methyl propionate | | GC-MS | **0,07** | 0,02 | 0,15 | **0,01** | 0,00 | 0,06 | 0,052 | 0,175 | -0,256 | 1,51 | | X |
| Ethyl propionate | | GC-MS | **3,31** | 1,42 | 5,02 | **1,90** | 0,59 | 4,20 | 0,330 | 0,566 | -0,129 | 1,03 | | X |
| Propyl propionate | | GC-MS | **0,43** | 0,16 | 1,52 | **0,33** | 0,10 | 1,57 | 0,755 | 0,843 | 0,042 | 1,09 | | X |
| Butyl propionate | | GC-MS | **0,04** | 0,00 | 0,33 | **0,00** | 0,00 | 0,25 | 0,755 | 0,843 | 0,042 | 1,50 | | X |
| Butyrate | | NMR | **10,01** | 8,51 | 11,71 | **9,22** | 7,18 | 10,40 | 0,018 | 0,100 | -0,312 | 1,41 | X | X |
| Butanoic acid | | GC-MS | **3,51** | 1,74 | 6,32 | **1,47** | 0,71 | 2,60 | 0,054 | 0,179 | -0,254 | 1,01 | | X |
| Methyl butyrate | | GC-MS | 0,89 | 0,28 | 3,38 | **0,22** | 0,06 | 0,72 | 0,022 | 0,108 | -0,301 | 1,39 | X | X |
| Ethyl butyrate | | GC-MS | **5,73** | 1,78 | 10,14 | **1,16** | 0,51 | 9,48 | 0,197 | 0,420 | -0,170 | 1,32 | | X |
| Propyl butyrate | | GC-MS | **2,99** | 0,43 | 8,35 | **0,83** | 0,20 | 4,99 | 0,428 | 0,645 | -0,105 | 1,16 | | X |
| Butyl butyrate | | GC-MS | **0,21** | 0,04 | 2,12 | **0,03** | 0,00 | 0,36 | 0,086 | 0,259 | -0,226 | 1,41 | | X |
| Ethyl valerate | | GC-MS | **1,71** | 0,39 | 4,97 | **0,71** | 0,37 | 3,60 | 0,874 | 0,938 | 0,021 | 1,69 | | X |
| Propyl valerate | | GC-MS | **0,27** | 0,11 | 0,71 | **0,20** | 0,06 | 0,69 | 0,982 | 0,988 | -0,004 | 1,08 | | X |
| Butyl valerate | | GC-MS | **0,03** | 0,00 | 0,10 | **0,00** | 0,00 | 0,08 | 1,000 | 1,000 | 0,000 | 1,46 | | X |
| Methyl valerate | | GC-MS | **0,36** | 0,13 | 0,94 | **0,18** | 0,05 | 0,72 | 0,367 | 0,606 | -0,119 | 1,24 | | X |
| Methyl caproate | MCFAs and MCFA derivatives | GC-MS | **0,11** | 0,00 | 0,83 | **0,00** | 0,00 | 0,19 | 0,141 | 0,344 | -0,194 | 1,16 | | X |
| Ethyl caproate | | GC-MS | **0,59** | 0,10 | 5,46 | **0,18** | 0,06 | 1,01 | 0,096 | 0,276 | -0,219 | 1,01 | | X |
| Propyl caproate | | GC-MS | **0,03** | 0,00 | 0,25 | **0,00** | 0,00 | 0,05 | 0,358 | 0,596 | -0,121 | 1,07 | | X |
| Heptanoic acid | | GC-MS | **0,13** | 0,04 | 0,26 | **0,05** | 0,00 | 0,32 | 0,547 | 0,727 | -0,080 | 1,14 | | X |
| Ethyl caprylate | | GC-MS | **0,04** | 0,00 | 0,31 | **0,00** | 0,00 | 0,04 | 0,047 | 0,175 | -0,262 | 1,24 | | X |

| | | | | | | | | | | | | | | |
|---|---|---|---|---|---|---|---|---|---|---|---|---|---|---|
| Methyl cyclohexanoate | carboxylic acid derivatives | GC-MS | **0,00** | 0,00 | 0,07 | **0,00** | 0,00 | 0,02 | 0,734 | 0,843 | -0,045 | 1,01 | | X |
| Ethyl cyclohexanoate | | GC-MS | **0,01** | 0,00 | 0,04 | **0,00** | 0,00 | 0,02 | 0,255 | 0,486 | -0,150 | 1,49 | | X |
| alpha-Ionone | sesquiterpene | GC-MS | **0,01** | 0,00 | 0,02 | **0,00** | 0,00 | 0,02 | 0,444 | 0,658 | -0,101 | 1,09 | | X |
| beta-Calacorene | | GC-MS | **0,01** | 0,00 | 0,02 | **0,01** | 0,00 | 0,01 | 0,846 | 0,919 | 0,026 | 1,45 | | X |
| Humulene | | GC-MS | **0,14** | 0,06 | 0,40 | **0,13** | 0,06 | 0,40 | 0,755 | 0,843 | 0,042 | 1,17 | | X |
| Caryophyllene | | GC-MS | **0,80** | 0,23 | 1,90 | **0,54** | 0,19 | 1,85 | 0,789 | 0,869 | -0,036 | 1,13 | | X |
| Terpinen-4-ol | monoterpene | GC-MS | **0,01** | 0,00 | 0,02 | **0,00** | 0,00 | 0,01 | 0,275 | 0,501 | -0,144 | 1,00 | | X |
| alpha-Phellandrene | | GC-MS | **0,18** | 0,09 | 0,60 | **0,15** | 0,04 | 0,31 | 0,466 | 0,679 | -0,096 | 1,04 | | X |
| 4-Ethyl-phenol | aromatic compounds | GC-MS | **0,04** | 0,01 | 0,36 | **0,01** | 0,00 | 0,04 | 0,039 | 0,160 | -0,271 | 1,56 | | X |
| o-Cymene | | GC-MS | **1,42** | 0,69 | 5,01 | **0,93** | 0,34 | 2,27 | 0,382 | 0,619 | -0,115 | 1,09 | | X |
| Cadalene | | GC-MS | **0,01** | 0,00 | 0,01 | **0,01** | 0,00 | 0,01 | 0,417 | 0,641 | 0,107 | 1,15 | | X |
| Isopropyl ethyl ketone | ketones | GC-MS | **0,61** | 0,45 | 1,05 | **0,42** | 0,20 | 0,69 | 0,220 | 0,442 | -0,162 | 1,09 | | X |
| 3-Octenone | | GC-MS | **0,05** | 0,01 | 0,09 | **0,01** | 0,00 | 0,06 | 0,118 | 0,304 | -0,206 | 1,10 | | X |
| unknown (RI 1136) | | GC-MS | **0,00** | 0,00 | 0,01 | **0,00** | 0,00 | 0,00 | 0,460 | 0,677 | -0,098 | 1,70 | | X |
| metabolite no. 58 | | NMR | **0,17** | 0,13 | 0,23 | **0,16** | 0,13 | 0,21 | 0,015 | 0,093 | -0,319 | 1,31 | X | X |
| metabolite no. 60 | | NMR | **0,05** | 0,04 | 0,06 | **0,04** | 0,03 | 0,05 | 0,017 | 0,100 | -0,313 | 1,46 | X | X |
| metabolite no. 59 | | NMR | **0,32** | 0,17 | 0,55 | **0,65** | 0,33 | 1,10 | 0,011 | 0,074 | 0,335 | 1,70 | X | X |
| p-Cresol | AAs and AA-fermentation products | GC-MS | **7,59** | 0,75 | 16,15 | **16,59** | 11,72 | 27,17 | 0,000 | 0,000 | 0,665 | 2,35 | X | X |
| Indole | | GC-MS | **5,10** | 2,32 | 10,33 | **9,94** | 7,81 | 18,60 | 0,000 | 0,001 | 0,588 | 2,18 | X | X |
| Scatole | | GC-MS | **0,40** | 0,13 | 2,70 | **4,78** | 0,51 | 12,05 | 0,001 | 0,012 | 0,451 | 1,73 | X | X |
| Methional | | GC-MS | **0,02** | 0,00 | 0,08 | **0,07** | 0,00 | 0,15 | 0,007 | 0,057 | 0,356 | | X | |
| aspartate | | GC-MS | **0,15** | 0,11 | 0,26 | **0,25** | 0,20 | 0,36 | 0,006 | 0,054 | 0,361 | 1,38 | X | X |
| lysine | | NMR | **0,23** | 0,17 | 0,37 | **0,35** | 0,30 | 0,51 | 0,184 | 0,398 | 0,175 | 1,20 | | X |
| Benzeneacetaldehyde | aromatic compounds | GC-MS | **0,67** | 0,28 | 1,16 | **1,22** | 0,52 | 1,81 | 0,001 | 0,018 | 0,431 | 1,67 | X | X |
| 2-pentyl thiophene | | GC-MS | **0,07** | 0,04 | 0,13 | **0,10** | 0,06 | 0,14 | 0,001 | 0,019 | 0,423 | 1,22 | X | X |
| Pentyl butyrate | SCFAs and SCFA-derivatives | GC-MS | **0,00** | 0,00 | 0,06 | **0,00** | 0,00 | 0,00 | 0,348 | 0,586 | 0,124 | 1,01 | | X |
| Valerate | | NMR | **0,19** | 0,10 | 0,30 | **0,29** | 0,24 | 0,37 | 0,027 | 0,120 | 0,292 | 1,39 | | X |
| Valeric acid | | GC-MS | **0,92** | 0,51 | 1,52 | **1,05** | 0,44 | 1,39 | 0,203 | 0,428 | 0,168 | 1,02 | | X |
| Isovalerate | BCFA | NMR | **1,30** | 0,67 | 2,12 | **1,93** | 1,28 | 2,63 | 0,143 | 0,344 | 0,193 | 1,07 | | X |

| | | | | | | | | | | | | | | |
|---|---|---|---|---|---|---|---|---|---|---|---|---|---|---|
| trimethylamine | | NMR | **0,15** | 0,11 | 0,18 | **0,20** | 0,17 | 0,27 | 0,271 | 0,499 | 0,145 | 1,35 | | X |
| Disulfide, dimethyl | | GC-MS | **0,23** | 0,06 | 0,54 | **0,33** | 0,12 | 0,65 | 0,051 | 0,175 | 0,257 | 1,12 | | X |
| 1-Pentanol | alcohol | GC-MS | **0,20** | 0,13 | 0,47 | **0,30** | 0,20 | 0,46 | 0,005 | 0,046 | 0,371 | 1,18 | X | X |
| 2-Nonanone | ketones | GC-MS | **0,17** | 0,09 | 0,28 | **0,22** | 0,12 | 0,32 | 0,009 | 0,066 | 0,345 | 1,15 | X | X |
| 2-Dodecanone | | GC-MS | **0,03** | 0,00 | 0,05 | **0,07** | 0,00 | 0,10 | 0,015 | 0,093 | 0,320 | | X | |
| 2-Undecanone | | GC-MS | **0,21** | 0,13 | 0,34 | **0,37** | 0,27 | 0,57 | 0,000 | 0,005 | 0,510 | 1,94 | X | X |
| 2-Tridecanone | | GC-MS | **0,21** | 0,12 | 0,32 | **0,35** | 0,15 | 0,54 | 0,000 | 0,008 | 0,477 | 1,35 | X | X |
| 2-Tetradecanone | | GC-MS | **0,03** | 0,00 | 0,06 | **0,06** | 0,02 | 0,09 | 0,003 | 0,032 | 0,396 | | X | |
| Dodecanal | aldehydes | GC-MS | **0,13** | 0,05 | 0,27 | **0,35** | 0,13 | 0,60 | 0,000 | 0,002 | 0,543 | 1,60 | X | X |
| Tetradecanal | | GC-MS | **0,08** | 0,05 | 0,15 | **0,12** | 0,04 | 0,39 | 0,002 | 0,031 | 0,401 | 1,51 | X | X |
| Pentadecanal- | | GC-MS | **0,06** | 0,02 | 0,13 | **0,07** | 0,03 | 0,17 | 0,011 | 0,074 | 0,335 | 1,08 | X | X |
| Hexadecanal | | GC-MS | **0,06** | 0,04 | 0,13 | **0,11** | 0,06 | 0,22 | 0,001 | 0,012 | 0,451 | | X | |
| Decane | alcanes | GC-MS | **0,18** | 0,06 | 0,29 | **0,20** | 0,11 | 0,37 | 0,011 | 0,074 | 0,336 | | X | |
| Dodecane | | GC-MS | **0,28** | 0,14 | 0,44 | **0,32** | 0,25 | 0,41 | 0,004 | 0,046 | 0,376 | 1,09 | X | X |
| Pentadecane | | GC-MS | **0,00** | 0,00 | 0,00 | **0,00** | 0,00 | 0,05 | 0,005 | 0,048 | 0,368 | 1,03 | X | X |
| Diallyl disulphide | | GC-MS | **0,03** | 0,00 | 0,09 | **0,07** | 0,02 | 0,13 | 0,003 | 0,035 | 0,390 | 1,15 | X | X |
| Carvone | monoterpenoid | GC-MS | **0,09** | 0,04 | 0,64 | **0,17** | 0,07 | 1,41 | 0,023 | 0,110 | 0,299 | 1,40 | | X |
| Unknown VOC (RI 1719) | | GC-MS | **0,06** | 0,00 | 0,09 | **0,10** | 0,03 | 0,17 | 0,005 | 0,046 | 0,374 | | X | |
| Unknown VOC (RI 1888) | | GC-MS | **0,08** | 0,03 | 0,18 | **0,12** | 0,04 | 0,24 | 0,031 | 0,131 | 0,285 | 1,05 | | X |
| Unknown VOC (RI 1897) | | GC-MS | **0,01** | 0,01 | 0,02 | **0,01** | 0,01 | 0,02 | 0,014 | 0,090 | 0,324 | | X | X |
| Unknown VOC (RI 1921) | | GC-MS | **0,11** | 0,04 | 0,23 | **0,24** | 0,12 | 0,35 | 0,000 | 0,008 | 0,480 | 1,25 | X | X |
| Unknown VOC (RI 1930) | | GC-MS | **0,04** | 0,02 | 0,06 | **0,09** | 0,03 | 0,15 | 0,001 | 0,012 | 0,452 | | X | |
| unknown VOC (RI 1993) | | GC-MS | **0,01** | 0,00 | 0,03 | **0,02** | 0,01 | 0,04 | 0,025 | 0,115 | 0,295 | 1,00 | | X |

Data are presented as compositional (proportion of total area under curve) and are shown as medians ($1^{st}$. $3^{rd}$ quartile). Only metabolites selected by at least one statistical methods as significantly contributing to the groups' separation are shown. Metabolites identified by NMR are given as normalized intensities (PQN normalized), VOCs are expressed as percent of the total fingerprint area. The scale of the values is comparable only among variables identified by the same method. Green lines indicates metabolites enriched in vegans, grey lines indicates metabolites depleted in vegans compared with omnivores. UDAA, univariable differential abundance analysis; FDR, false discovery rate; PLS-DA, partial least squares discriminant analysis; VIP, variable importance in projection. VIP score of a variable measures its discriminatory power and is computed as root of sum of weighted explained sum of squares over all dimensions, divided by the cumulative explained sum of squares.

**Table S7** *Bile acids in feces*

| | | VG | O | p value | FDR |
|---|---|---|---|---|---|
| secondary BAs | LCA (nM) | 5.9 ± 0.8 | 9.5 ±1.1 | 0.003 | 0.034 |
| | DCA (nM) | 2.4 ± 0.9 | 4.4 ± 0.8 | n.s. | |
| | UDCA (nM) | 0.04 ± 0.01 | 0.07 ± 0.02 | n.s. | |
| | gDCA (nM) | 0.02 ± 0.01 | 0.03 ± 0.01 | n.s. | |
| | tDCA (nM) | 0.01 ± 0.01 | 0.01 ±0.01 | n.s. | |
| | tLCA (nM) | 0.01 ± 0.01 | 0.01 ± 0.01 | n.s. | |
| primary BAs | CDCA (nM) | 0.16 ± 0.05 | 0.18 ± 0.07 | n.s. | |
| | CA (nM) | 0.11 ± 0.03 | 0.10 ± 0.04 | n.s. | |
| | gCDCA (nM) | 0.02 ± 0.01 | 0.04 ± 0.02 | n.s. | |
| | gCA (nM) | 0.02 ± 0.01 | 0.03 ± 0.01 | n.s. | |
| | tCDCA (nM) | 0.01 ± 0.01 | 0.01 ± 0.01 | n.s. | |

**Table S8** *Serum metabolome composition*

| name | detection method | | VG | | | O | | | p-value | FDR | delta | VIP | UDAA | PLS-DA |
|---|---|---|---|---|---|---|---|---|---|---|---|---|---|---|
| | | | median | Q1 | Q3 | median | Q1 | Q3 | | | Cliff´s | | | |
| Glycine | NMR | non-essential AA | 4,93 | 4,36 | 5,76 | 3,60 | 3,15 | 4,15 | 0,000 | 0,000 | -0,683 | 1,55 | X | X |
| Glutamine | NMR | | 16,67 | 15,87 | 17,73 | 15,63 | 14,53 | 16,24 | 0,000 | 0,000 | -0,635 | 1,66 | X | X |
| Asparagine | NMR | | 0,42 | 0,37 | 0,49 | 0,37 | 0,32 | 0,41 | 0,000 | 0,000 | -0,522 | 1,56 | X | X |
| Proline | NMR | | 4,62 | 4,34 | 5,10 | 4,50 | 4,28 | 4,77 | 0,018 | 0,030 | -0,295 | | X | |
| Threonine | NMR | essential AA | 1,52 | 1,40 | 1,66 | 1,45 | 1,32 | 1,55 | 0,012 | 0,024 | -0,316 | | X | |
| Butyric acid | LC-MS | SCFA | 0,20 | 0,12 | 0,31 | 0,11 | 0,06 | 0,16 | 0,000 | 0,000 | -0,519 | 1,12 | X | X |
| Propionic acid | LC-MS | | 0,25 | 0,15 | 0,47 | 0,15 | 0,08 | 0,32 | 0,000 | 0,000 | -0,505 | 1,29 | X | X |
| Formic acid | LC-MS | | 74,24 | 66,34 | 79,42 | 70,16 | 61,11 | 78,02 | 0,000 | 0,001 | -0,468 | 1,03 | X | X |
| Acetic acid | LC-MS | | 23,57 | 18,92 | 31,17 | 27,91 | 19,69 | 36,31 | 0,006 | 0,014 | -0,344 | | X | |
| Acetate | NMR | | 1,98 | 1,69 | 2,43 | 1,65 | 1,46 | 1,89 | 0,001 | 0,002 | -0,429 | | X | |
| Citrate | NMR | tricarboxylic acid | 0,66 | 0,58 | 0,79 | 0,63 | 0,53 | 0,70 | 0,015 | 0,026 | -0,305 | | X | |
| Dimethylsulfone | NMR | | 1,32 | 1,16 | 1,43 | 1,14 | 1,10 | 1,30 | 0,001 | 0,002 | -0,431 | | X | |
| Histidine | NMR | essential AA | 0,83 | 0,76 | 0,88 | 0,92 | 0,84 | 1,00 | 0,013 | 0,025 | 0,311 | | X | |
| Tryptophane | NMR | | 1,22 | 1,12 | 1,32 | 1,33 | 1,26 | 1,44 | 0,001 | 0,004 | 0,400 | | X | |
| Lysine | NMR | | 2,19 | 1,99 | 2,40 | 2,72 | 2,52 | 2,84 | 0,000 | 0,000 | 0,680 | 1,72 | X | X |
| Valine | NMR | essential AA; BCAA | 6,10 | 5,55 | 6,69 | 7,43 | 6,73 | 7,95 | 0,000 | 0,000 | 0,568 | 1,45 | X | X |
| Leucine | NMR | | 5,70 | 5,13 | 6,30 | 6,69 | 6,31 | 6,96 | 0,000 | 0,000 | 0,541 | 1,45 | X | X |
| Isoleucine | NMR | | 1,36 | 1,27 | 1,54 | 1,53 | 1,43 | 1,68 | 0,011 | 0,023 | 0,320 | 1,46 | X | X |
| 2-hydroxybutyrate | NMR | BCAA derivative | 0,94 | 0,80 | 1,12 | 1,22 | 1,04 | 1,41 | 0,000 | 0,000 | 0,510 | 1,20 | X | X |
| 2-oxoisocaproate | NMR | | 0,46 | 0,39 | 0,56 | 0,55 | 0,51 | 0,66 | 0,002 | 0,006 | 0,384 | 1,43 | X | X |
| 2-oxoisovalerate | NMR | | 0,56 | 0,47 | 0,64 | 0,66 | 0,59 | 0,71 | 0,004 | 0,011 | 0,356 | 1,31 | X | X |
| 3-hydroxy-isobutyrate | NMR | | 0,33 | 0,24 | 0,40 | 0,38 | 0,33 | 0,44 | 0,014 | 0,026 | 0,308 | | X | |
| Valeric acid | LC-MS | SCFA | 0,01 | 0,00 | 0,02 | 0,02 | 0,01 | 0,04 | 0,017 | 0,029 | 0,297 | 1,13 | X | X |
| metabolite no. 63 | NMR | | 0,41 | 0,34 | 0,45 | 0,49 | 0,43 | 0,54 | 0,000 | 0,000 | 0,480 | 1,29 | X | X |

Data are presented as normalized intensities and are shown as medians (1st. 3rd quartile). Only metabolites selected by at least one statistical methods as significantly contributing to the groups' separation are shown. Metabolites identified by NMR are given as normalized intensities (PQN normalized), SCFA concentrations are given in µM. The scale of the values is comparable only among variables identified by the same method.   Green lines indicates metabolites enriched in vegans, grey lines indicates metabolites depleted in vegans compared with omnivores. UDAA, univariable differential abundance analysis; FDR, false discovery rate; PLS-DA, partial least squares discriminant analysis; VIP, variable importance in projection. VIP score of a variable measures its discriminatory power and is computed as root of sum of weighted explained sum of squares over all dimensions, divided by the cumulative explained sum of squares.

**Table S9** *Urine metabolome composition*

| | detection method | VG | | | O | | | p-value | FDR | Cliff´s delta | VIP | UDAA | PLS-DA |
|---|---|---|---|---|---|---|---|---|---|---|---|---|---|
| | | median | Q1 | Q3 | median | Q1 | Q3 | | | | | | |
| Trigonelline | NMR | 0,40 | 0,23 | 0,51 | 0,22 | 0,10 | 0,43 | 0,014 | 0,035 | -0,319 | | X | |
| Glycine | NMR | 8,24 | 6,39 | 14,16 | 6,05 | 3,84 | 7,55 | 0,038 | 0,075 | -0,269 | | X | |
| Creatinine | NMR | 46,84 | 39,85 | 54,58 | 51,76 | 46,49 | 56,04 | 0,000 | 0,003 | 0,453 | 1,46 | X | X |
| 3-Indoxylsulfate | NMR | 0,06 | 0,04 | 0,09 | 0,09 | 0,07 | 0,13 | 0,001 | 0,003 | 0,442 | 1,28 | X | X |
| Methylnicotinamide | NMR | 0,08 | 0,06 | 0,11 | 0,10 | 0,08 | 0,14 | 0,000 | 0,002 | 0,470 | 1,41 | X | X |
| 3-hydroxy Isobutyrate | NMR | 0,15 | 0,11 | 0,19 | 0,19 | 0,14 | 0,24 | 0,000 | 0,002 | 0,492 | 1,59 | X | X |
| 3-hydroxy Isovalerate | NMR | 1,10 | 0,88 | 1,38 | 1,29 | 1,06 | 1,57 | 0,005 | 0,017 | 0,366 | 1,27 | X | X |
| Trimethylamino oxid | NMR | 13,97 | 11,49 | 18,61 | 15,08 | 12,54 | 26,18 | 0,008 | 0,025 | 0,341 | | X | |
| Valine | NMR | 0,12 | 0,09 | 0,16 | 0,13 | 0,11 | 0,15 | 0,029 | 0,065 | 0,283 | | X | |
| p-Cresyl sulfate | NMR | 1,20 | 0,59 | 2,04 | 1,44 | 1,14 | 2,13 | 0,043 | 0,078 | 0,261 | | X | |
| 2-hydroxy Isobutyrayte | NMR | 0,18 | 0,15 | 0,22 | 0,19 | 0,17 | 0,26 | 0,118 | 0,152 | 0,202 | 1,15 | | X |
| Dimethylamine | NMR | 0,88 | 0,75 | 1,07 | 0,98 | 0,82 | 1,12 | 0,102 | 0,152 | 0,212 | 1,05 | | X |

Data are presented as normalized intensities and are shown as medians (1st, 3rd quartile). Only metabolites selected by at least one statistical methods as significantly contributing to the groups' separation are shown. Green lines indicates metabolites enriched in vegans, grey lines indicates metabolites depleted in vegans compared with omnivores. UDAA, univariable differential abundance analysis; FDR, false discovery rate; PLS-DA, partial least squares discriminant analysis; VIP, variable importance in projection. VIP score of a variable measures its discriminatory power and is computed as root of sum of weighted explained sum of squares over all dimensions, divided by the cumulative explained sum of squares.

**Supplemental figures**

**Figure S1** *Representative ¹H NMR spectrum of serum with quantified metabolites*. Metabolite assignments for the numbers are given in Table S1.

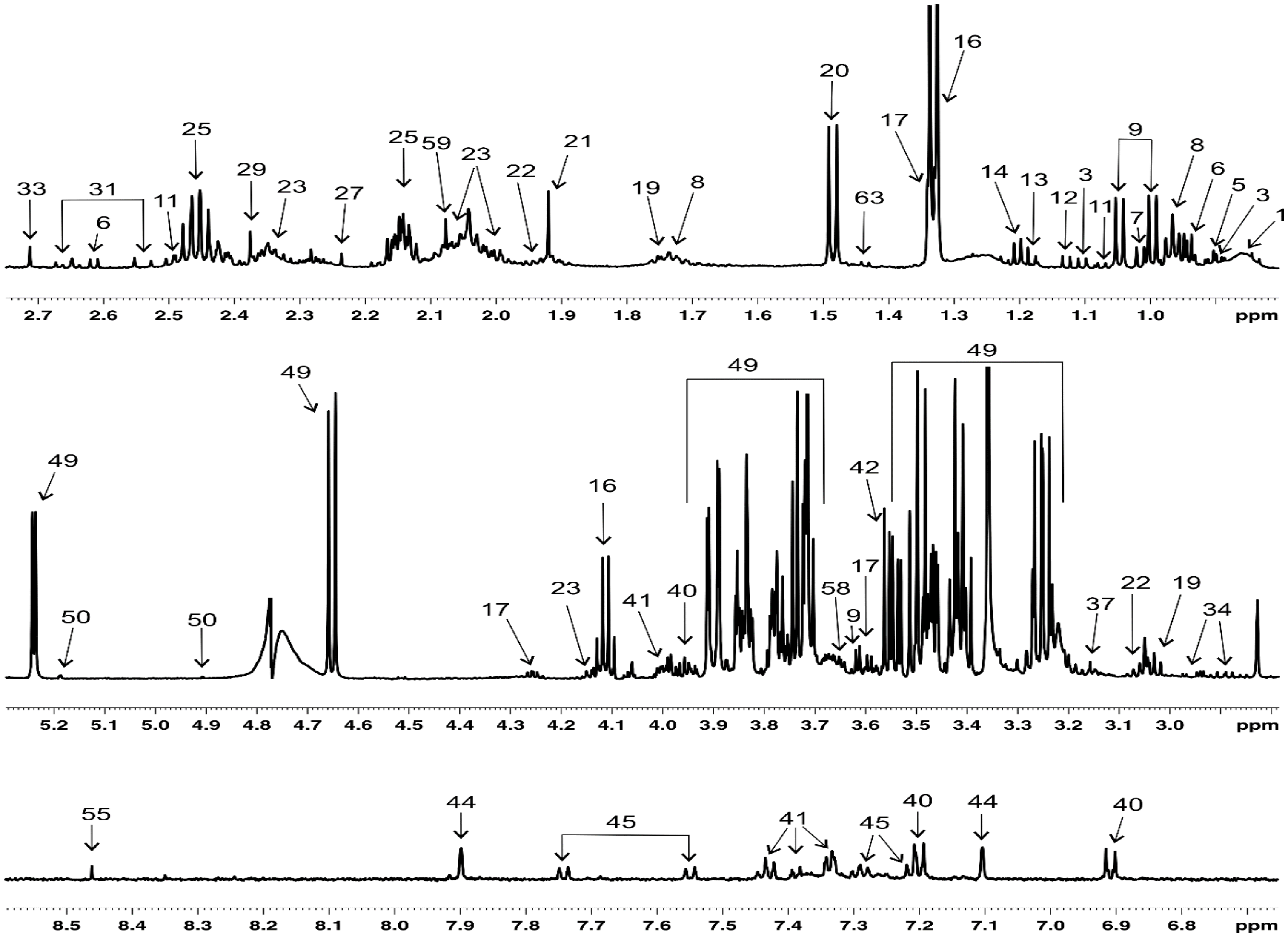

**Figure S2** *Representative $^{1}$H NMR spectrum of fecal extract with quantified metabolites*. Metabolite assignments for the numbers are given in Table S1.

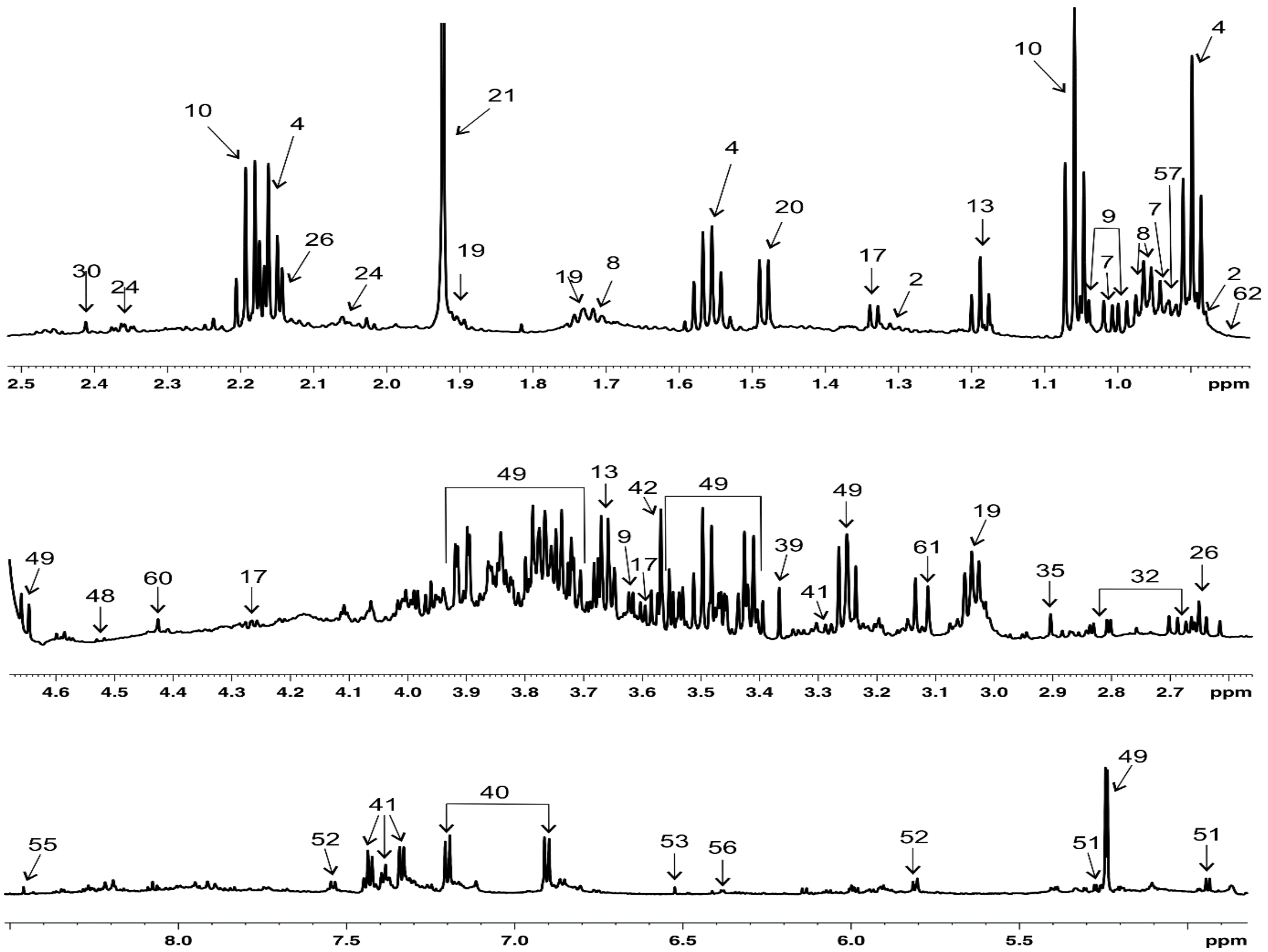

**Figure S3** *Representative $^{1}$H NMR spectrum of urine with quantified metabolites*. Metabolite assignments for the numbers are given in Table S

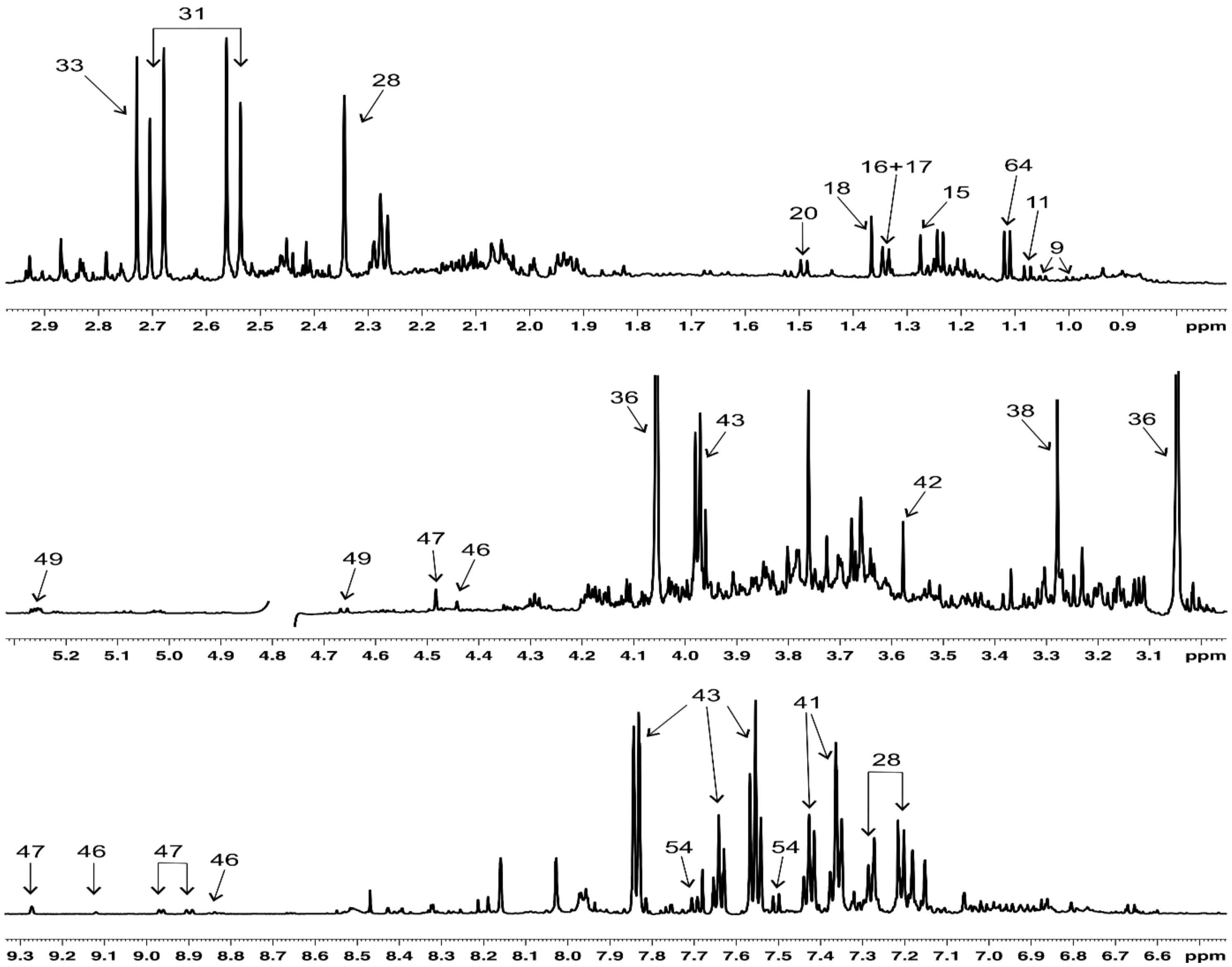

Supplement: Supplementary file 1 [file Data_Sheet_1.PDF]
